# Multivariate Entropy Characterizes the Gene Expression and Protein-Protein Networks in Four Types of Cancer

**DOI:** 10.3390/e20030154

**Published:** 2018-02-28

**Authors:** Angel Juarez-Flores, Marco V. José

**Affiliations:** 1 Posgrado en Ciencias Biológicas, Unidad de Posgrado, Circuito de Posgrados, Ciudad Universitaria, Universidad Nacional Autónoma de México, CP 04510, Mexico City, Mexico; 2 Theoretical Biology Group, Instituto de Investigaciones Biomédicas, Universidad Nacional Autónoma de México, CP 04510, Mexico City, Mexico

**Keywords:** multivariate entropy, cancer, protein-protein networks, gene expression, local networks, average network entropy, biomarkers, early warning

## Abstract

There is an important urgency to detect cancer at early stages to treat it, to improve the patients’ lifespans, and even to cure it. In this work, we determined the entropic contributions of genes in cancer networks. We detected sudden changes in entropy values in melanoma, hepatocellular carcinoma, pancreatic cancer, and squamous lung cell carcinoma associated to transitions from healthy controls to cancer. We also identified the most relevant genes involved in carcinogenic process of the four types of cancer with the help of entropic changes in local networks. Their corresponding proteins could be used as potential targets for treatments and as biomarkers of cancer.

## 1. Introduction

Cancer is a generic term given to a collection of related diseases that can arise in every part of the organism. Typically, there is an increased division rate, dysregulation in growth and the capacity to spread into surrounding tissues and eventually on distant tissues. The latter process is known as metastasis, and is the main cause of death by cancer [1,2]. Entropy can be defined as the measure of the average uncertainty of a single random variable in bits, whereas the differential entropy is the entropy of a continuous random variable with an important characteristic that it only depends on the probability density of the random variable [3]. Unlike discrete entropy, the differential entropy can be negative [3]. Differential entropy can be conceived as the logarithm of the equivalent side length of the smallest set that contains most of the probability. Hence, low entropy implies that the random variable is confined to a small effective volume and high entropy indicates that the random variable is widely dispersed [3]. Entropy can be used as a descriptive and comprehensive measure of multivariate variability especially when data are non-Gaussian, since it can capture higher-order statistics and information content of the data [4]. 

Liver cancer is one of the main causes of cancer-related deaths worldwide [5]. Hepatocellular carcinoma (HCC) is the most common type of primary liver cancer and the third most common cause of cancer-related deaths [6,7]. Its incidence and mortality continue to rise, chronic viral hepatitis and cirrhosis being some risk factors. Early screening has a survival benefit for patients. Available methods for HCC screening are radiographic, but unfortunately diagnosis is often made at advanced states of the disease, when effectiveness of treatment has poor prognosis. Sorafenib is the recommended treatment with patients in advanced stages, but due to its side effects it is difficult for the patient to tolerate it [8]. It is a disease with 10 to 20% recurrence even in patients with liver transplantation, which is the most successful treatment [6]. There was a previous work in which entropy was used to detect an early warning in HCC, where the transition occurs at a very early stage of HCC [9]. 

Pancreatic cancer is one of the deadliest types of cancer. It was reported as the fourth cause of death cancer-related in developed countries in 2012. The GLOBOCAN estimations of new cases were 94,700 and 92,800 and estimated deaths were 93,100 and 91,300, in males and females, respectively. In 2015 for ductal adenocarcinoma, the most common type of pancreatic cancer, 367,000 new cases were diagnosed, and 359,000 patients died that same year. A pancreatic adenoma is followed by a neoplasm which leads to a carcinoma [10]. For diagnosis, there is currently no proper biomarker with enough specificity and sensitivity, yet the detection of the earliest stages of pancreatic cancer are urgently needed to improve the outcomes of resection, which is so far, the best treatment. Surgery is the only option for cure but only 10–15% of the newly diagnosed patients are eligible [11]. Due to the resistance of pancreatic cancer to therapy, even with resection, most of the patients will relapse and eventually succumb to the disease [12].

Lung cancer has the highest mortality rate. The rate of survival of patients with lung cancer is less than 5% after five years, and it tends to metastasize, thereby early diagnosis is important. Squamous cell carcinoma represents approximately 30% of all the cases [13,14]. Usually the stages of carcinogenesis are: squamous metaplasia → dysplasia → carcinoma in situ → squamous cell carcinoma of lung [15,16]. Squamous cell carcinoma (SCC) of the lung is the most common histologic subtype of non-small cell lung cancer (NSCLC). It accounts for 400,000 new cases annually worldwide, with cigarette smoking as the principal risk factor. For advanced stages, the standard care consists of a platinum-based doublet as a palliative systemic therapy [17]. Diagnosis is made by histological analysis of small biopsies or cytological specimens as fine needle aspirates or bronchial brushings. There are new promising therapies that improve patient’s survival [14].

Melanoma is only one among various dermatological cancers, but it is the principal cause of death due to skin cancer, accounting for approximately 80%, and with an increasing incidence in the last years with sun exposure as the main risk factor [18,19]. All melanomas originate from melanocytes which represents a minority of the cell population within the basilar epidermis, but it can also be found in hair follicles and other tissues. Melanocytes provide the pigment melanin to their neighboring keratinocytes. Pigment production is stimulated by UV radiation-induced DNA damage to keratinocytes [20]. The gold standard for diagnosis is histopathological assessment [19]. There is an ideal model of melanoma carcinogenesis ranging from benign naevi → dysplastic naevi → melanoma in situ → invasive melanoma [20]. 

Teschendorff et al. [21] proposed signaling entropy as a novel approach for analyzing and interpreting omics data, such as discriminating cells according to their differentiation potential and cancer status. In other works, some driver genes were found to be associated with reductions in network entropy [9,21,22]. More recently, Brehme et al. [23] carried out an analysis in chronic myeloid leukemia using entropy dynamics and separated the progression stages. They revealed an important difference in the chronic phase (CP) which allowed to separate it into two phases: “early” from “late” CP [23]. That same year Park et al. [24], using an entropy-based distance metric, were able to successfully measure the intratumor heterogeneity and propose it as a useful tool to characterize it at the RNA level using transcriptome and network information.

Cancer research has a wide variety of approaches, mainly for therapeutics usage, ranging from analysis of massive data, such as the genome or transcriptome, to more detailed analyses as of single genes or proteins that are involved in cancer hallmarks [25]. A major challenge is the opportune detection of the early stages of the disease, which is the most desirable scenario, with better options for patients treatment and an improved outcome as seen in pancreatic cancer and lung cancer, where cancer is commonly detected in the last stages of the disease [12,13,14]. Finding new therapeutics targets are important due to cancer resistance to therapies and to have more repertoires of targets that could be modulated by immunotherapy, miRNAs therapy, gene therapy, or other treatments [26,27].

In general, cancer progression can be divided into three states [9]: a normal state, a pre-disease state (or a critical state), and a disease state. In the normal state, the disease is under control (immune system) and dynamically it has high resilience and robustness to perturbations. The pre-disease state is defined as the limit of the normal state, which occurs before the imminent phase transition point is reached, but it has low resilience and robustness due to its dynamical structure [9]. The disease state represents a seriously deteriorated stage possibly with high resilience and robustness, where the system usually finds it difficult to recover or return to the normal state even after treatment, which contrasts with the pre-disease state. Therefore, it is crucial to detect the pre-disease state to prevent qualitative deterioration and to further elucidate its molecular mechanism. In cancer, it is a daunting task to predict a pre-disease state because the state of the system may change little before the bifurcation point or the critical transition is reached. There may be slight differences between the normal and pre-disease. The detection of early-warning signals can involve a myriad of genetic factors. There are leading networks in critical transitions, which make the first move from a normal state to a disease state [9]. The leading network is the first subnetwork that breaks down the limit of a normal state to move into a disease state, which means that they are clearly related to the causal or driving genes in a disease network, in contrast to the differential gene expression that results from the disease. Therefore, identifying the leading networks during a critical transition can signal the emergence of a pre-disease state to make the early diagnosis on the disease, and help to disentangle the mechanisms of disease initiation and progression at the network level. The leading networks are dynamical signals that herald the pre-disease state, rather than the disease state detected by the traditional static biomarkers.

Herein, we contend that multivariate entropy is a useful filter for detecting driver genes. Therefore, we calculated the multivariate entropy of the gene expression profiles in four types of cancer, to wit, pancreatic cancer, melanoma, HCC, and squamous cell carcinoma of the lung. For these cancers, we also constructed their corresponding protein-protein interaction (PPI) networks considering the disease stages. We calculated the multivariate entropy for the local networks of PPI from which we estimated the average entropy of PPI networks. In general, the reliable identification of local leading networks and pre-disease stages is not easy to achieve with noisy data and a small number of samples. The identification of biomarkers and the critical states may be inaccurate. In this work, we validated our proposed biomarkers using different statistical tests, such as double filter for the differentially gene expression, from which we selected the genes for constructing the local networks. The Wilcoxon rank sum test was used to test the statistical differences in the average network entropy between the pre-disease and diseased states with the normal state. We searched the biological function of those genes whose entropy changed and some of them are already considered potential therapeutic targets, for example see [28]. With our analyses, we also found new potential targets whose biological functions are relevant to the normal cell function. We successfully identified the most relevant genes involved in the carcinogenic processes of the four types of cancer with the help of entropic changes in local networks. Their corresponding proteins could be used as potential targets for treatments and as biomarkers of cancer.

## 2. Materials and Methods

Four series of raw transcriptomic data of the carcinogenesis process were retrieved from the Gene Expression Omnibus (GEO) of the National Center for Biotechnology Information (NCBI). The first one: hepatocellular carcinoma with GEO accession: GSE6764, which has 75 tissue samples (platform: Affymetrix Human Genome U133 Plus 2.0 Array [29]). The second: melanoma, GEO accession: GSE4587, with 18 samples (platform: Affymetrix Human Genome U133 Plus 2.0 Array [30]). The third: pancreatic cancer, GEO accession: GSE19650, with 22 samples (platform: Affymetrix Human Genome U133 Plus 2.0 Array [31]). The fourth: squamous cell carcinoma of the lung, GEO accession: GSE33479, with 122 samples (platform: Agilent-014850 Whole Human Genome Microarray 4 × 44 K G4112F).

We used the R software with Limma package [32] according to its manual and the Bioconductor Manual to preprocess and process all the transcriptomic data. Only melanoma and squamous cell carcinoma of the lung were retrieved preprocessed (background corrected, normalized) from GEO. The Limma package was used for differentially gene expression analysis obtaining the respective adjusted *p*-value for False Discovery Rate (*FDR*) [32,33]. Differential gene expression analysis was made between each stage and the normal one. Fold changes were also calculated, and we used a double filter to select the differentially expressed genes. Criteria consist in selecting genes with an adjusted *p*-value < 0.05 and Fold Change > 1.5 [32,33,34,35]. To create the networks of Protein-Protein interactions (PPIs) of each cancer, we used the APID database [36] which provides protein interaction data for a wide variety of species with a controlled quality using PPI found by experimental evidence. We used data set from quality level 1 (all known interactions). Herein, we used the *Homo sapiens* data which were processed and cleaned using the Cytoscape Software version 3.2 [37]. Cleanup consisted in the deletion of data of other species based on its taxonomic tag, and in the deletion of duplicated edges and nodes. The proteins retrieved from the database were the ones detected by the double filter applied to the differentially gene expression but due to the lack of information of the Human interactome, we only retrieved at least 50% of the proteins coded by the genes detected by the double filter analysis. Then, we obtained the first neighbors for each node in the network using Rcy3 [38] which allows a connection between R and Cytoscape. The result was used to create the *local networks* of each node (protein). We tested the hypothesis that the distribution of expression levels across all the selected genes for the four cancers followed a normal or a log-normal distribution (Appendix A) [39]. The density function of the multivariate normal distribution is given by [3,38]:

(1) $$f(x)=\frac{1}{|2\pi \Sigma {|}^{1/2}}\exp\{-\frac{1}{2}{\left(x-\mu \right)}^{\prime }\Sigma^{-1}(x-\mu )\},$$

where *x* is a random vector, *μ* is the mean vector, and Σ is the covariance matrix.

In each stage, the local networks data were matched with their respective gene expression of each sample to create matrices of *local networks* where the genes expression were the rows and each column a sample. For each *local network* matrix, a covariance matrix was calculated and then we applied Equation (2) to obtain the multivariate entropy of each *local network*. Each sample is a set of vectors $X_{1}X_{2},$ and so on. The expression level of the genes are elements of the vectors.

We also group nodes (proteins) to create subnetworks. Based on the maximum and minimum calculated entropy values of the healthy states, we established the limit from where a preset range was applied as follows: HCC in ranges of 10 units resulting in 11 subnetworks, Melanoma in ranges of three units resulting in eight subnetworks, Pancreatic in ranges of five units resulting in nine subnetworks, Squamous cell lung carcinoma in ranges of 10 units resulting in 19 subnetworks. The starting point was the 0 unit. This healthy-generated groups were kept in the four types of cancer resulting in that each successive stage has the same groups with same nodes with a unique variation in their entropy values. To calculate the entropy for each local network, we consider the entropy of multivariate normal distribution given by [3,40]:

(2) $$h(X_{{}_{1}},X_{2},\ldots ,X_{n})=h(N_{n}(\mu ,K))=\frac{1}{2}\log_{2}{\left(2\pi e\right)}^{n}|\kappa |,$$

where |κ| is the determinant of the covariance matrix; *X* is the random variable (set of vectors); *n* is the size of the covariance matrix. 

Entropy of a local network is calculated with Equation (3), where a single value is calculated from the data with all genes within the same local network, and colored rectangles (Supplementary Materials) represent the entropy change for the *local network*.

The average network entropy, *H*(*t*) is given by:

(3) $$H(t)=-\frac{1}{n}\sum_{i=1}^{n}h_{i}(t),$$

where *n* is the number of nodes in the network (differentially expressed genes) and *h_i_*(*t*) is the entropy of the local network *i*. Equation (3) is the same as the one used in [9], but with a negative sign to obtain positive values of entropy and to have units of information in bits.

Gene expression entropies (Figure 2, Tables 1–4) were calculated using all selected genes by the double filter to create a single matrix for every stage in each cancer, without using the *local networks* data. The level expression of the genes are elements of the vectors. From the matrix, a covariance matrix was calculated and then Equation (2) was applied to obtain the gene expression entropies.

For each entropy calculation, the following number of samples in each stage were used: HCC: seven samples; melanoma: two samples; pancreatic cancer: three samples; squamous cell lung cancer: 12 samples. The identification was made by looking at the color changes in the nodes. Colors represent a range of entropy values, so if a node has a change in color from one to another we record it.

### 2.1. Statistical Analysis of Local Network Entropy

Wilcoxon Rank Sum test was used to compare the results of local network entropy in each stage of the carcinogenic process versus its respective control for the four types of cancers (see Appendix A). Calculations were made with the R package. This probe is a non-parametric statistical test to compare two population medians, and it allows to determine if two probes have significant differences. 

### 2.2. Local Networks

**Figure 1:**
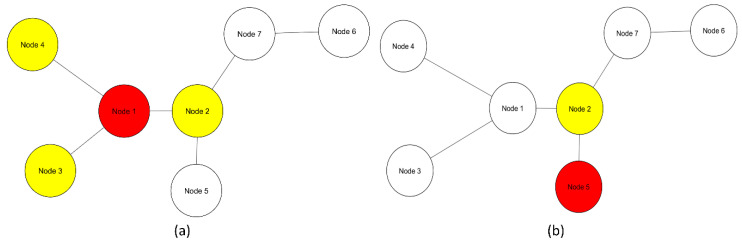
Graph of basic network with seven nodes. (**a**) A local network of node 1 (red) consists of its first neighbors (yellow) and node 1; (**b**) Local network of node 5 (red) is constituted by its first, and in this case, only neighbor node 2 (yellow) and node 5 itself.

**Figure 2:**
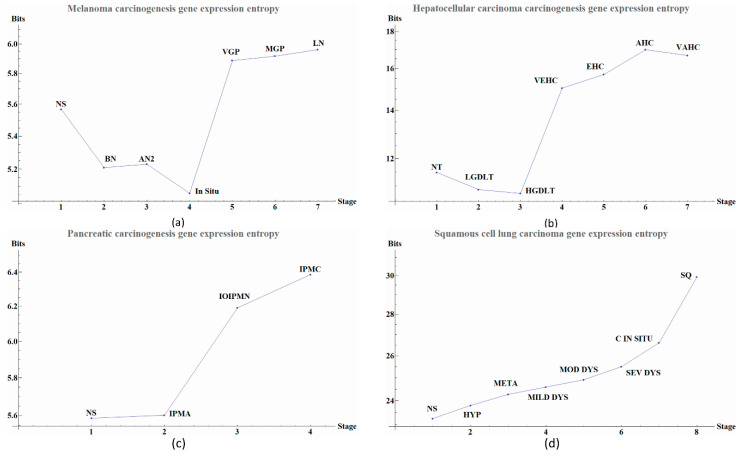
Gene expression-entropy of four types of cancer. Scale of the axes vary depending upon the type of cancer and sample size. (**a**) Melanoma carcinogenesis gene expression-entropy. Each point in the graph denote a stage during carcinogenic process the last four points denote cancer and the previous are pre-cancerous stage. The meanings of the abbreviated labels are given in Table 1. (**b**) Hepatocellular carcinoma carcinogenesis gene expression-entropy. Each point in the graph denote a stage during carcinogenic process the last four points denote cancer and the previous are pre-cancerous stage. The labels abbreviations are given in Table 2. (**c**) Pancreatic carcinogenesis gene expression-entropy. Each point in the graph denote a stage during carcinogenic process the last point denote cancer and the previous are pre-cancerous stage. The label abbreviation meanings are given in Table 3. (**d**) Squamous cell lung carcinoma carcinogenesis gene expression-entropy. Each point in the graph denote a stage during carcinogenic process the two points denote cancer and the previous are pre-cancerous stage. The label abbreviation meanings are given in Table 4.

Multivariate entropy of gene expression profiles is influenced only by gene expression (Figure 2). Multivariate entropy of local networks of PPIs is also influenced by gene expression with the addition of another level of complexity: the PPIs of each node. Entropy values of local networks will be influenced by which nodes constitute the local network and their respective gene expression values (Figure 1)

## 3. Results

Herein we illustrate how entropy changes throughout the distinct stages of the carcinogenesis process in the four types of cancer. We used Equation (2) to obtain the global change in entropy using the gene expression of the selected genes by the double filter criteria. We found that each cancer stage displays a characteristic entropic value when compared with pre-cancerous stages.

We observe that the last part of the carcinogenesis processes, the gene expression possesses the largest positive entropy than all previous stages with the only exception of melanoma in situ. The latter could be ascribed to different pathways and inherent heterogeneities among the different regions of the body where biological carcinogenesis of melanoma ensues (Figure 2 and Table 1, Table 2, Table 3 and Table 4) [20]. In Figure 2a we observed variations in entropy values throughout all carcinogenic process of Melanoma, with the most evident change occurring at in situ stage in which its entropy value reaches a minimum. This change can be used as a first cancer early warning. Notice in Figure 2b that in HCC carcinogenic process the entropy values in pre-cancerous stages were decreasing until a sudden change in the transition point from a pre-cancerous to cancer stage. This change is in agreement with a first cancer early warning [9]. In Figure 2c we observe for pancreatic cancer that entropy increases as the carcinogenic process progresses. A sudden change occurs between intraductal papillary-mucinous adenoma and intraductal papillary-mucinous neoplasm stages that are not cancer stages and therefore we need to be careful to talk about a cancer early warning. In Figure 2d we observe that for squamous cell lung carcinoma, entropy is increasing gradually in the precancerous stages. The last stages correspond to cancer and they appear to have a greater change in entropy with an obvious change in entropy between carcinoma in situ and the squamous cell carcinoma of the lung. The change between severe dysplasia and carcinoma in situ could be a cancer early warning. 

To find out which genes had major changes in every stage and how they are related between them, we constructed a protein-protein network for each carcinogenic process and assigned their respective local entropies for each stage. Then we calculated the average network entropy using the local entropy values and built groups based in health controls and for each stage of the carcinogenic process. The PPIs networks of melanoma are displayed in Figure 3. The PPIs networks with detail of melanoma, HCC, pancreatic cancer and squamous cell carcinoma of the lung are displayed in Supplementary Materials (Figures S1–S26) The entropy values were ranked and graded by colors. Notice that color variations in each group correspond to variations in entropy.

**Figure 3:**
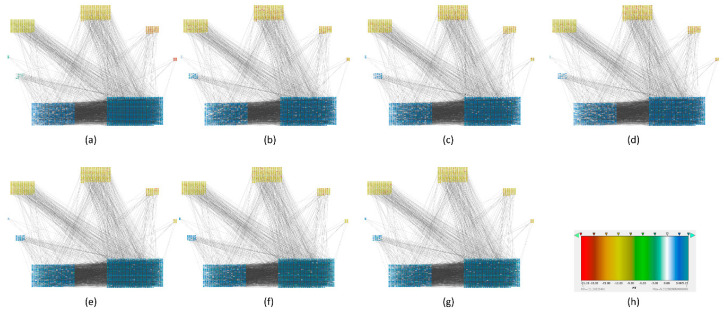
Melanoma carcinogenesis PPIs with local entropy. PPIs networks in which every node has an associated value that correspond to its local network-entropy (**a**) Normal PPIs network (**b**) Benign nevi PPIs network (**c**) Atypical nevi PPIs network (**d**) In situ melanoma PPIs network (**e**) Vertical growth phase melanoma PPIs network (**f**) Metastatic growth phase melanoma PPIs network (**g**) Lymph node metastasis PPIs network (**h**) Represent scale of entropy and the color assigned to every group.

**Figure 4:**
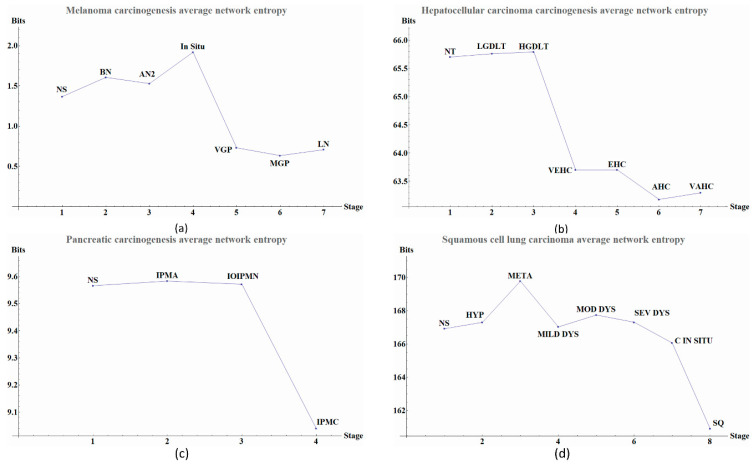
Average network entropy by local network of four types of cancer. Scale of the axes vary according to the type of cancer and sample size. (**a**) Melanoma carcinogenesis average network entropy by local networks. Graph shows entropy values in each stage. Each point in the graph denotes a stage during carcinogenic process the last four points denote cancer and the previous are pre-cancerous stage. The label abbreviation meanings are given in Table 5 with their respective value for each stage. (**b**) Hepatocellular carcinoma carcinogenesis average network entropy by local networks. Graph shows entropy values in each stage. Each point in the graph denotes a stage during carcinogenic process the last four points denote cancer and the previous are pre-cancerous stage. The meanings of the label abbreviations are in given in Table 6 with their respective value for each stage. (**c**) Pancreatic carcinogenesis average network entropy by local networks. Graph shows entropy values in each stage. Each point in the graph denotes a stage during the carcinogenic process. The last point denotes cancer and the previous points are pre-cancerous stages. The abbreviation meanings are given in Table 7 with their respective value for each stage. (**d**) Squamous cell lung carcinoma average network entropy by local networks. Graph shows entropy values in each stage. Each point in the graph denotes a stage during carcinogenic process the last two points denote cancer and the previous are pre-cancerous stage. The abbreviation meanings are given in Table 8 with their respective values for each stage. Some descriptive statistics as the interquartile range, standard error and median about average network entropy are found in Appendix A (Table A1, Table A2, Table A3 and Table A4). Some characteristics of *local networks* of each stage, as the number of local networks and size of them between groups, are found in Appendix B (Table A13, Table A14, Table A15 and Table A16).

The calculated network entropy of PPIs and the entropy values calculated from expression data are positive (Figure 2 and Figure 4). The average network entropy of local networks of melanoma and HCC exhibit a concave pattern in its entropy values of only gene expression, although the magnitudes are greater for HCC than for melanoma possibly due to the number of samples in each case. The interesting behaviors of the calculated average network entropy are seen in Figure 4c,d. In Figure 4c of the pancreatic carcinogenic process, we can identify an early warning due to a sudden change observed between the non-cancer to cancer stage which is statistically significant by Wilcoxon rank sum test with continuity correction (*p*-value = 4.537 × 10^−7^, see Appendix A), albeit this was not observed with its entropy values from only gene expression. Something similar occurs with Figure 2d, in which there is a smooth tendency of increasing entropy as the process progresses, but this pattern is not the same with the one observed in Figure 3d. In this case, there is an evident variation among stages with three major changes in metaplasia with an increased entropy value, carcinoma in situ stage with a slight decrease compared with SQ in which entropy decreases drastically and this change is statistically significant (calculated *p*-value = 0.0001274, see Appendix A). The melanoma carcinogenic process (Figure 3a) also denotes variations. There is an important change, melanoma in situ stage increases its entropy and the following stages decrease it and these changes are statistically significant (*p* = 2.2 × 10^−16^, see Appendix A). No statistically significant changes for HCC were found (not shown). Dunnett’s test were applied for each cancer with no statistically significant results (Appendix A, Table A5, Table A6, Table A7 and Table A8). For these cases, the average network entropy is useful as a first observation, whereas the most important data come from the local network entropy. Local network entropy values permit us to dissect each cancer to visualize the most important variations at each stage.

Some genes like CYP2C9, FDX1, MUT, VAMP4, IL33, EMP2, DENND4A have drastic changes in its entropy during the transition from atypical nevi to melanoma in situ, as observed in Figure S10 and S11 (see Supplementary Materials). In the case of IL33, which is implicated in maturation of Th2 cell and the activation of MPK signaling pathway through IL1RL1/ST2 receptor and this pathway improves cell proliferation [41]. Previous studies tried to associate this protein with cancer promotion, but different results were found [28]. CYP2C9 polymorphisms were associated with colorectal cancer risk [42] and may influence breast cancer [43]. EMP2 is a protein implicated in progression and survival in endometrial cancer, and recently it was proposed as a possible oncoprotein [44]. We highlight that there are not studies in cancer for FDX1, MULT, VAMP4 and DENND4A genes or its protein products.

We found some genes in the transition of HGDLT to VECH in HCC like SOX6, ASPH, UBAP2L, CEP41. SOX6 encodes a transcription factor with a key role in developmental processes and it has been associated to HCC progression by its decreased progression [45]. UBAP2L was associated with the metastatic ability in some HCC cell lines via SNAIL1 [46]. Recently ASPH was suggested as a potential biomarker in gliomas [47]. There are no studies of the role of CEP41 in cancer. In pancreatic cancer, the transition between IOIPMN to IPMC includes the following genes: FAR1, CEACAM1, HCCS. CEACAM1 has 11 different splice variants, as reported in some studies in vivo, and restoration of its expression abolishes oncogenicity of tumor cell lines, but when it is expressed de novo it increases the risk of metastasis [48] CEACAM1 has also been proposed as a potential biomarker for breast cancer [49]. There are no studies for HCCS and FAR1 in cancer. In the transition of squamous severe dysplasia to carcinoma in situ, the genes UBET2 PIH1D2, KIF23 showed a change in their entropy. KIF23 is a protein essential for cytokinesis in Rho-mediated signaling. Its overexpression is associated with lung cancer cell growth and has been suggested as a novel therapeutic target for patients with advanced lung cancer and primary lung tumors [50]. A recent study showed that a knockdown of UBET2 induced an inhibition in the progression of gastric cancer in vivo and in vitro via WNT signal pathway [51]. There are no studies of PIH1D2 in cancer. A summary of the proposed genes for each cancer is shown in Table 9.

## 4. Discussion

In this work, we have succeeded in the identification of the most relevant genes involved in carcinogenic processes of four types of cancer with the help of entropic changes in local networks. We are validating the use of multivariate entropy by testing that the distributions of gene expressions can follow either a normal distribution (SCC of the lung and melanoma) or a log-normal distribution (HCC and pancreatic cancer) (Appendix A).

We found that cancer entropic values from the average network entropy were lower than the observed values in healthy controls, which agrees with previous analysis [21,22]. The entropic values of the networks at the final stages for all examined cancers fully comply with the latter observation. However, not all pre-advanced cancer stages followed this behavior as we observed in melanoma in situ where entropy is higher than the control. Overall, entropy values correlate to each cancer stage. Interestingly, entropy values from only gene expression have a different behavior than those from the PPIs. In some cases, they could look like a mirror image of each other as is the case of HCC, but this is not always the case as was observed in pancreatic cancer or squamous cell carcinoma of the lung. As we mentioned previously, the differential entropy can be negative [3].

Our analysis of the carcinogenic processes allowed us to identify initial stages of the four types of cancer at which entropy changed with respect the control. A similar early warning measured with entropy for the average network was observed in HCC but this change was not statistically significant [9]. In pancreatic cancer, however, we found that the sudden change in average network entropy is statistically significant.

We also characterized each stage by local network entropy of its genes, which permitted to identify the most important genes in each stage and the ones in the transition between them. We illustrated the transitions from pre-cancer to cancer stage in which some of the found genes have been previously reported and even proposed to be early biomarkers in cancer by experiments in vivo and/or in vitro [47,49]. We also proposed new genes as biomarkers and as potential therapeutic targets (Table 1) that have not been previously reported: for melanoma: FDX1 (essential for the synthesis of various steroid hormones [41]), MUT (involved in degradation of several amino acids, odd-chain fatty acids and cholesterol via propionyl-CoA to the tricarboxylic acid cycle [41]), VAMP4 (involved in the pathway that functions to remove an inhibitor of calcium-triggered exocytosis during the maturation of secretory granules [41]), DENND4A (promotes the exchange of GDP to GTP, converting inactive GDP-bound Rab proteins into their active GTP-bound form [41]); for HCC: CEP41 (required during ciliogenesis for tubulin glutamylation in cilium [41]); for pancreatic cancer: FAR1 (catalyzes the reduction of saturated and unsaturated C16 or C18 fatty acyl-CoA to fatty alcohols [41]) and HCCS (stress-activated component of a protein kinase signal transduction cascade and regulates the JNK and p38 pathways [41]); for squamous cell carcinoma of the lung: PIH1D2 (exhibits a Ral GTPase binging which means a selectively interacting and non-covalently with Ral protein [41]). Our proposed genes as potential biomarkers or therapeutic targets for melanoma and pancreatic cancer must be taken with caution due to their small sample sizes. Due to its biological relevance, we hope that our results of local networks strongly inspire further experimental work for testing the proposed genes as biomarkers and/or therapeutic targets. In the Supplementary Materials, we provide entropic values for all the local networks of genes from healthy stage to all stages of cancer. This work was focused only in protein coding genes and the PPIs of its products, that represent less than 1.5% of the human genome [52]. There is a wide field of cancer research such as non-coding-DNA/RNA, single nucleotide polymorphisms, copy number variations, and epigenetic factors such as methylation and acetylation, that could lead us to a better understanding of the dynamics of cancer diseases. 

## Figures and Tables

**Table 1:** Melanoma carcinogenesis process and their respective entropy values.

Abbreviation	Stage	Entropy Value
NS	Normal skin	5.5685
BN	Benign nevi	5.2100
AN2	Atypical nevi	5.2303
In situ (INS)	Melanoma in situ	5.0595
VGP	VGP melanoma Vertical growth phase melanoma	5.8874
MGP	MGP melanoma Metastatic growth phase melanoma	5.9169
LN	Lymph node metastasis	5.9612

**Table 2:** Hepatocellular carcinoma carcinogenesis process and their respective entropy values.

Abbreviation	Stage	Entropy Value
NT	Normal skin	11.4778
LGDLT	Low grade dysplasia	10.8693
HGDLT	High grade dysplasia	10.7351
VEHC	Very early hepatocellular carcinoma	15.0311
EHC	Early hepatocellular carcinoma	15.6988
AHC	Advanced hepatocellular carcinoma	16.9940
VAHC	Very advanced hepatocellular carcinoma	16.6807

**Table 3:** Pancreatic carcinogenesis process and their respective entropy values.

Abbreviation	Stage	Entropy Value
NS	Normal main pancreatic duct	5.5868
IPMA	Intraductal papillary-mucinous adenoma	5.6022
IOIPMN	Intraductal papillary-mucinous neoplasm	6.1912
IPMC	intraductal papillary-mucinous carcinoma	6.3856

**Table 4:** Squamous carcinogenesis process and their respective entropy values.

Abbreviation	Stage	Entropy Value
NS	Normal	23.2530
HYP	Hyperplasia	23.8010
META	Metaplasia	24.2762
MILD DYS	Mild dysplasia	24.5936
MOD DYS	Moderate dysplasia	24.9167
SEV DYS	Severe dysplasia	25.5035
C IN SITU	Carcinoma in situ	26.6175
SQ	Squamous cell carcinoma	29.9367

**Table 5:** Melanoma carcinogenesis process and their respective average network entropy values from PPIs.

Abbreviation	Stage	Entropy Value
NS	Normal skin	1.3677
BN	Benign nevi	1.6061
AN2	Atypical nevi	1.5287
INS	Melanoma in situ	1.9190
VGP	VGP melanoma Vertical growth phase melanoma	0.7308
MGP	MGP melanoma Metastatic growth phase melanoma	0.6339
LN	Lymph node metastasis	0.7081

**Table 6:** Hepatocellular carcinogenesis process and their respective average network entropy values from PPIs.

Abbreviation	Stage	Entropy Value
NT	Normal skin	65.7023
LGDLT	Low grade	65.7600
HGDLT	High grade	65.7916
VEHC	Very early HCC	63.7020
EHC	Early HCC	63.7023
AHC	Advanced HCC	63.1783
VAHC	Very advanced HCC	63.2960

**Table 7:** Pancreatic cancer carcinogenesis process and their respective average network entropy values from PPIs.

Abbreviation	Stage	Entropy Value
NS	Normal main pancreatic duct	9.5664
IPMA	Intraductal papillary-mucinous adenoma	9.5835
IOIPMN	Intraductal papillary-mucinous neoplasm	9.5720
IPMC	Intraductal papillary-mucinous carcinoma	9.0398

**Table 8:** Squamous cell lung carcinoma carcinogenesis process and their respective average network entropy values from PPIs.

Abbreviation	Stage	Entropy Value
NS	Normal	166.9220
HYP	Hyperplasia	167.3054
META	Metaplasia	169.7816
MILD DYS	Mild dysplasia	167.0286
MOD DYS	Moderate dysplasia	167.7428
SEV DYS	Severe dysplasia	167.3106
C IN SITU	Carcinoma in situ	166.0658
SQ	Squamous cell carcinoma	160.9167

**Table 9:** Proposed genes as potential biomarkers or therapeutic targets

Cancer	Proposed Genes
Melanoma	FDX1, MUT, VAMP4, DENND4A
HCC	CEP41
Pancreatic cancer	FAR1, HCCS
Squamous cell carcinoma of the lung	PIH1D2

**Figure A1:**
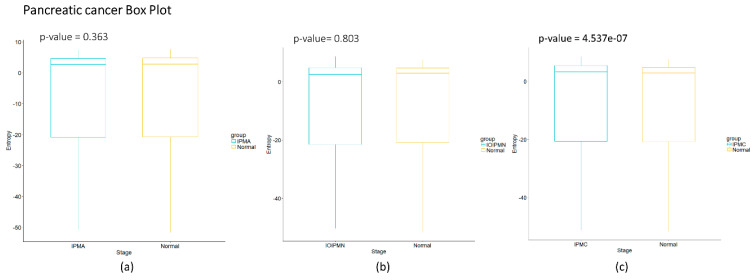
Pancreatic carcinogenesis process boxplot. The graph shows the comparison of the different stages vs. the control with boxplot and a *p*-value corresponding to the Wilcoxon rank sum test. (**a**) Intraductal papillary-mucinous adenoma; (**b**) Intraductal papillary-mucinous neoplasm; (**c**) Intraductal papillary-mucinous carcinoma.

**Figure A2:**
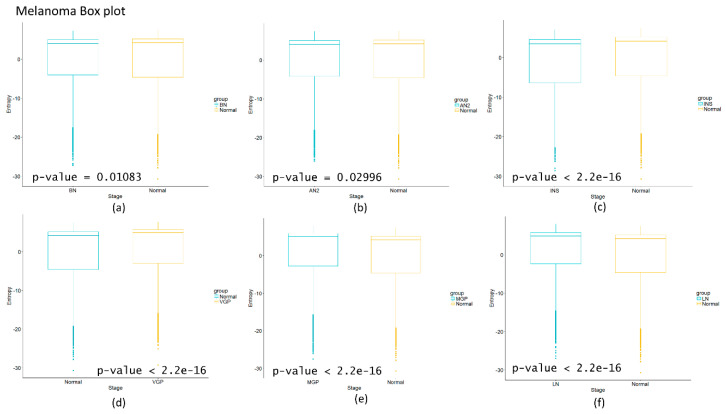
Melanoma carcinogenesis process boxplot. The graph shows the comparison of the different stages vs. the control with boxplot and a *p*-value corresponding to the Wilcoxon rank sum test. (**a**) Benign nevi; (**b**) Atypical nevi; (**c**) In situ melanoma; (**d**) Vertical growth phase melanoma; (**e**) Metastatic growth phase melanoma; (**f**) Lymph node metastasis.

**Figure A3:**
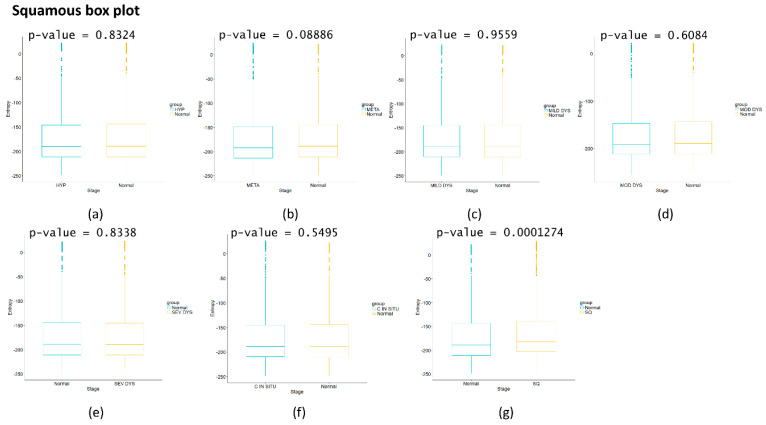
Squamous carcinogenesis process boxplot. The graph shows the comparison of the different stages vs. the control with boxplot and a *p*-value corresponding to the Wilcoxon rank sum test. (**a**) Hyperplasia; (**b**) Metaplasia; (**c**) Mild displasia; (**d**) Moderate displasia; (**e**) Severe Dysplasia; (**f**) Carcinoma in situ; (**g**) Squamous cell carcinoma.

**Figure A4:**
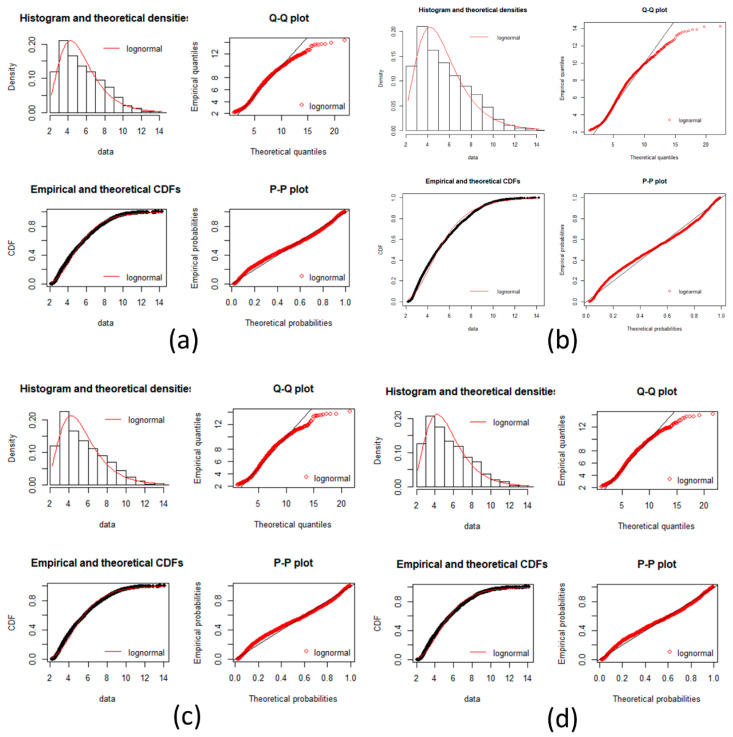
Pancreatic cancer goodness of fit test plot. Plots of goodness fits test for a log-normal distribution is showed in each incise for gene expression data. Histogram, Q-Q plot, CDFs and P-P plots are common plots to test a fitness of distribution. It can be seen that each stage fit well for a log normal distribution. (**a**) Normal stage; (**b**) IPMA stage; (**c**) IOIPMN stage; (**d**) IPMC stage.

**Figure A5:**
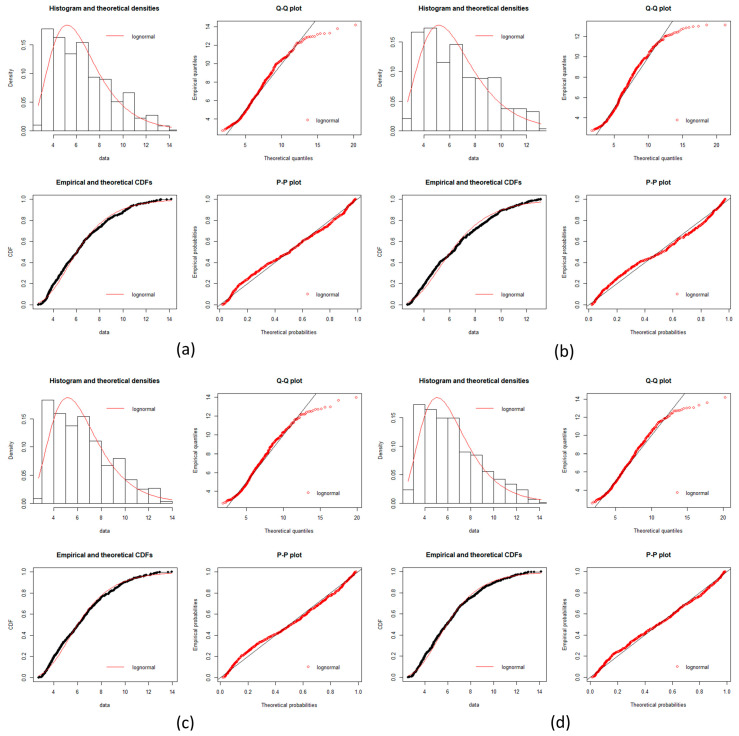
Hepatocellular carcinoma goodness of fit test plots part 1. Plots of goodness fits test for a log-normal distribution is showed in each incise for gene expression data. Histogram, Q-Q plot, CDFs and P-P plots are common plots to test a fitness of distribution. It can be seen that each stage fit well for a log normal distribution. (**a**) Normal stage; (**b**) LGDLT stage; (**c**) HGDLT stage; (**d**) VEHC stage.

**Figure A6:**
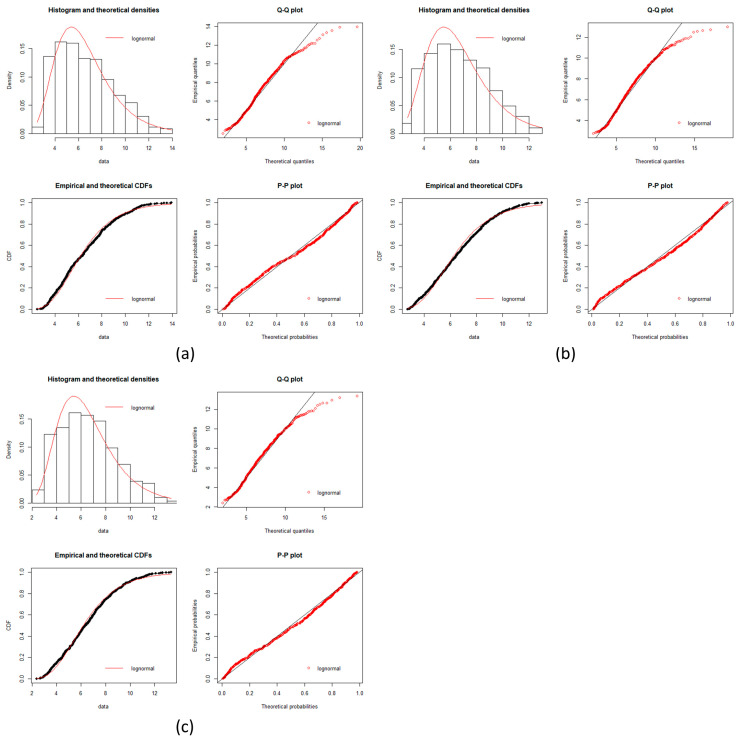
Hepatocellular carcinoma goodness of fit test plot part 2. Plots of goodness fits test for a log-normal distribution is showed in each incise for gene expression data. Histogram, Q-Q plot, CDFs and P-P plots are common plots to test a fitness of distribution. It can be seen that each stage fit well for a log normal distribution. (**a**) EHC; (**b**) AHC; (**c**) VAHC.

**Figure A7:**
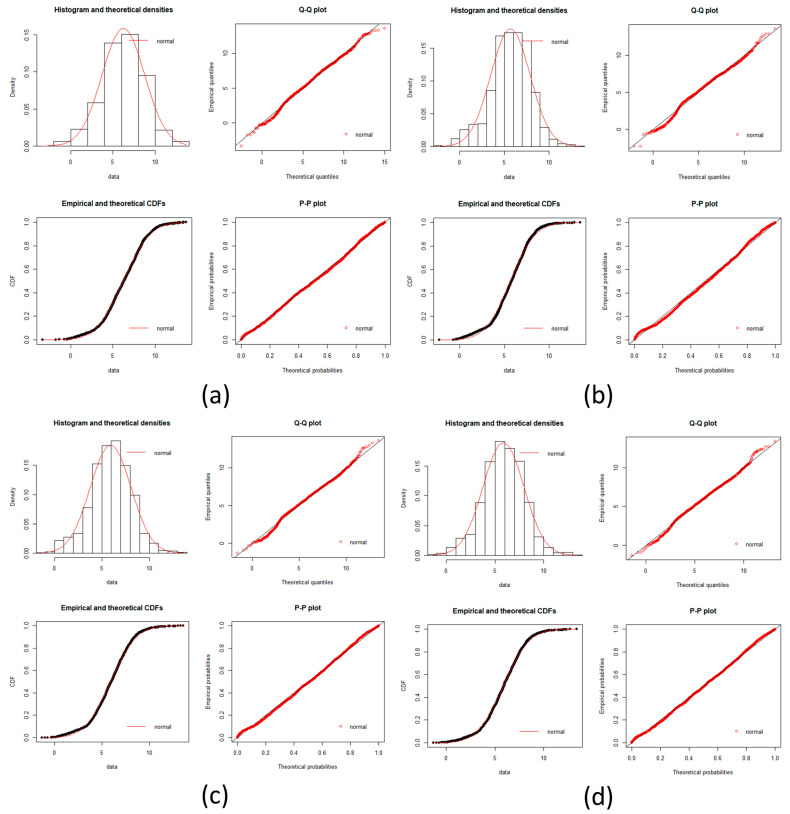
Melanoma goodness of fit test plot part 1. Plots of goodness fits test for a log-normal distribution is showed in each incise for gene expression data. Histogram, Q-Q plot, CDFs and P-P plots are common plots to test a fitness of distribution. It can be seen that each stage fit well for a log normal distribution. (**a**) Normal stage; (**b**) BN stage; (**c**) AN2 stage; (**d**) INS stage.

**Figure A8:**
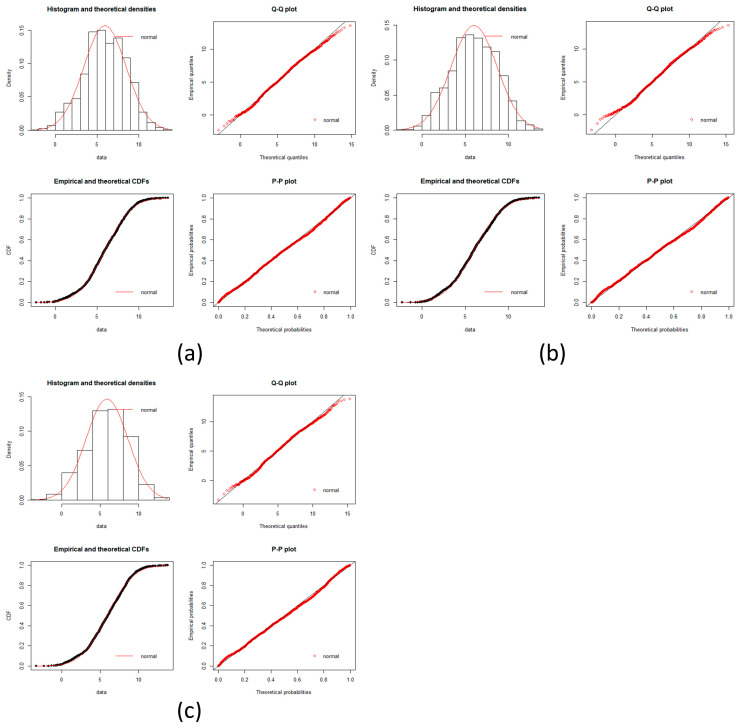
Melanoma goodness of fit test plot part 2. Plots of goodness fits test for a log-normal distribution is showed in each incise for gene expression data. Histogram, Q-Q plot, CDFs and P-P plots are common plots to test a fitness of distribution. It can be seen that each stage fit well for a log normal distribution. (**a**) VGP stage; (**b**) MGP stage; (**c**) LN stage.

**Figure A9:**
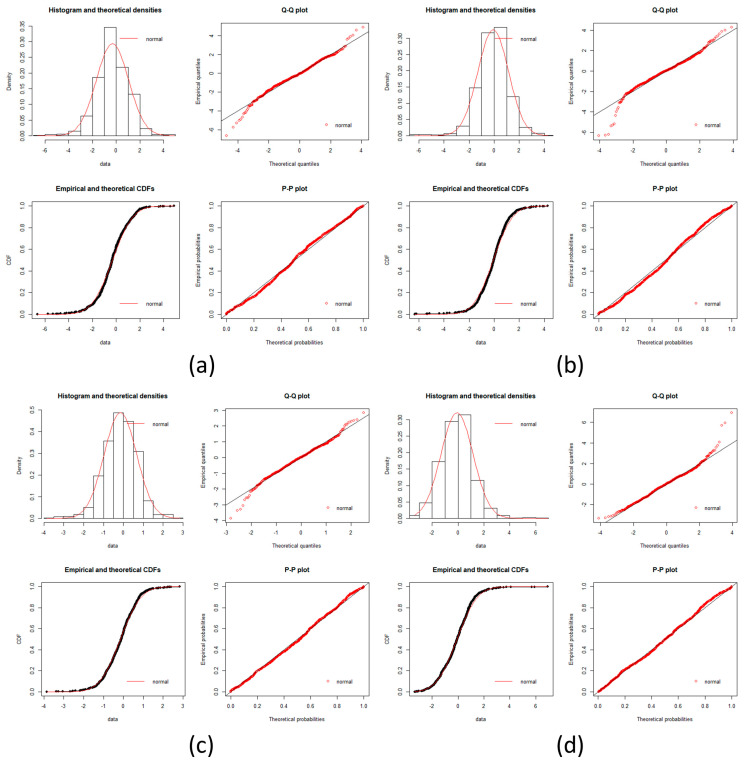
Squamous cell carcinoma of the lung goodness of fit test plot part 1. Plots of goodness fits test for a log-normal distribution is showed in each incise for gene expression data. Histogram, Q-Q plot, CDFs and P-P plots are common plots to test a fitness of distribution. It can be seen that each stage fit well for a log normal distribution. (**a**) Normal stage; (**b**) HYP stage; (**c**) META stage; (**d**) MILD DYS stage.

**Figure A10:**
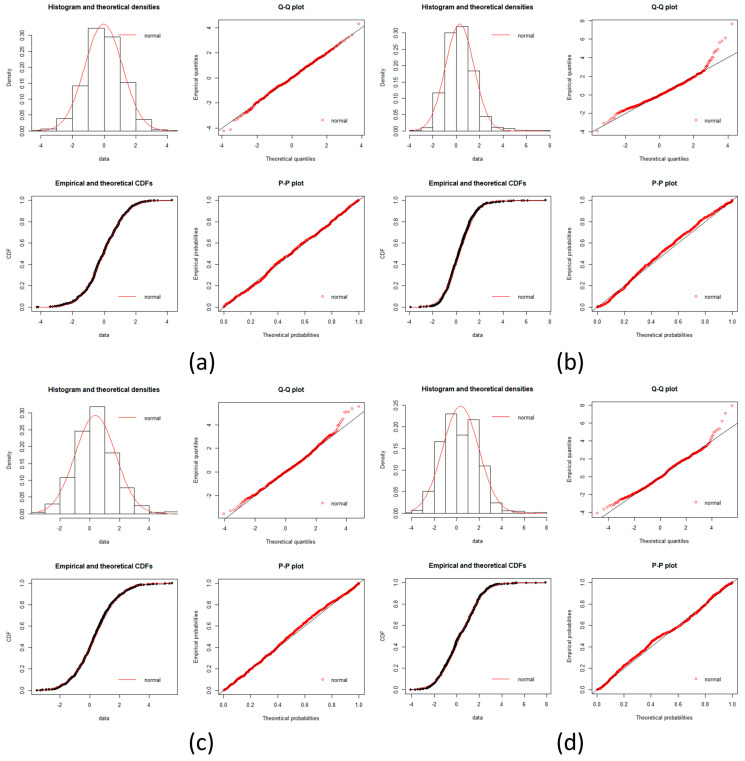
Squamous cell carcinoma of the lung goodness of fit test plot part 2. Plots of goodness fits test for a log-normal distribution is showed in each incise for gene expression data. Histogram, Q-Q plot, CDFs and P-P plots are common plots to test a fitness of distribution. It can be seen that each stage fit well for a log normal distribution. (**a**) MOD DYS stage; (**b**) SEV DYS stage; (**c**) C IN SITU stage; (**d**) SQ stage.

## Supplementary Materials

The following are available online at http://www.mdpi.com/1099-4300/20/3/154/s1.

## Appendix A. Statistical Analyses

### Appendix A.1. Descriptive Statistics for the Average Network Entropy of Each Stage in the Four Types of Cancer

Statistics estimations were made using the R software and we used the following packages: bootstrap, plotrix, dplyr [53,54,55].

**Table A1:** Melanoma statistics.

Stage	Mean	Standard Error	MAD	Median	IQR	Jack.se When Applied to Mean	Jack.se When Applied to Variance
Normal	−1.367775	0.2259220	2.022601	4.154535	9.776100	0.225922	2.814388
BN	−1.606108	0.2303059	2.001127	4.019503	9.047087	0.2303059	2.896202
AN2	−1.528727	0.2292635	1.941641	4.050469	9.248341	0.2292635	2.849997
INS	−1.919017	0.2262065	2.086082	3.455508	10.95625	0.2262065	2.813802
VGP	−0.7308951	0.2282546	1.690730	4.886908	8.654485	0.2282546	2.815323
MGP	−0.6339521	0.2299159	1.659544	5.048833	8.677472	0.2299159	2.873484
LN	−0.7081158	0.2259220	2.022601	4.154535	9.77610	0.2281064	2.805363

MAD: Median Absolute Deviation, IQR: Interquartile Range. Jack.se: Jackknife standard error.

**Table A2:** Pancreatic cancer statistics.

Stage	Mean	Standard Error	MAD	Median	IQR	Jack.se When Applied to Mean	Jack.se When Applied to Variance
Normal	−9.566407	0.4283494	3.899366	2.901261	25.45503	0.4283494	8.116081
IPMA	−9.583562	0.4229802	4.222622	2.726233	25.45141	0.4229802	7.979798
IOIPMN	−9.572038	0.4243606	4.743738	2.520672	26.37048	0.4243606	7.967539
IPMC	−9.039864	0.4288906	4.647821	3.350183	26.11237	0.4288906	8.078527

MAD: Median Absolute Deviation, IQR: Interquartile Range. Jack.se: Jackknife standard error.

**Table A3:** Squamous cell carcinoma statistics.

Stage	Mean	Standard Error	MAD	Median	IQR	Jack.se When Applied to Mean	Jack.se When Applied to Variance
NS	−166.9220	2.07917	37.68617	−189.2606	67.45387	2.07917	242.6845
HYP	−167.3055	2.07897	35.41448	−190.7849	65.29746	2.07897	244.6484
META	−169.7816	2.075519	3538473	−192.9719	64.74367	2.075519	247.9728
MILD DYS	−167.0286	2.078943	37.16981	−189.2085	65.54929	2.078943	245.2105
MOD DYS	−167.7429	2.083012	34.75794	−191.3746	64.21930	2.083012	248.3033
SEV DYS	−167.3107	2.082539	35.95474	−189.9894	65.61071	2.082539	247.5942
C IN SITU	−166.0658	2.079962	36.71084	−188.7635	64.23117	2.079962	247.5762
SQ	−160.9168	2.05378	36.48999	−182.8355	63.88627	2.05378	238.1753

MAD: Median Absolute Deviation, IQR: Interquartile Range. Jack.se: Jackknife standard error.

**Table A4:** HCC statistics.

Stage	Mean	Standard Error	MAD	Median	IQR	Jack.se When Applied to Mean	Jack.se When Applied to Variance
NT	−45.54143	1.322529	37.53280	−51.37492	62.92547	1.908006	74.42024
LGDLT	−45.58139	1.319797	37.67159	−51.74393	62.86078	1.904064	74.35039
HGDLT	−45.60330	1.319819	37.25316	−5189218	63.02723	1.904096	74.39116
VEHC	−44.15488	1.353999	39.67826	−50351675	64.91867	1.953408	77.8041
EHC	−44.15512	1.353269	38.7171	−51.31032	64.92842	1.952355	77.72415
AHC	−43.79192	1.377034	40.60674	−50.27078	67.11002	1.98664	80.5827
VAHC	−43.87345	1.370975	40.65539	−50.52407	66.22477	1.977898	79.51231

MAD: Median Absolute Deviation, IQR: Interquartile Range. Jack.se: Jackknife standard error.

### Appendix A.2. Dunnett’s Test for the Mean

Dunnett’s test was applied to compare the means of each stage vs. the control for the four types of cancer and effect size is also reported. No significant statistical results were found.

Cohen’s formula was used in the calculation of effect size:

$$d=M_{1}-M_{2}/s_{pooled}$$

where:

*M*_1_ − *M*_2_ is the difference between the group mean (M)
*M*_1_ is the mean of the control group
*M*_2_ is the mean of either group that is not the control
*s* is the pooled standard deviation:

$$s_{pooled}=\sqrt{\frac{\left(SD_{1}^{2}+SD_{2}^{2}\right)}{2}}$$

where:

*SD*_1_ is the standard deviation of the control group
*SD*_2_ is the mean of either group that is not the control

**Table A5:** Melanoma Dunnett Contrasts and effect size.

Hypotheses	*t*-Value	Pr(>|t|)	Effect Size
Normal-BN	−0.738	0.947	−0.0233
Normal-AN2	−0.499	0.992	−0.0158
Normal-INS	−1.707	0.333	−0.0544
Normal-VGP	1.973	0.202	0.0626
Normal-MGP	2.273	0.105	0.07191
Normal-LN	2.043	0.175	0.0649

**Table A6:** Pancreatic cancer Dunnett Contrasts and effect size.

Hypotheses	*t*-Value	Pr(>|t|)	Effect Size
Normal-IPMA	−0.028	1.000	−0.000915
Normal-IOIPMN	−0.009	1.000	−0.000299
Normal-IPMC	0.874	0.713	0.027897

**Table A7:** Squamous cell carcinoma of the lung Dunnett Contrasts and effect size.

Hypotheses	*t*-Value	Pr(>|t|)	Effect Size
Normal-HYP	−0.131	1.000	−0.00629
Normal-META	−0.974	0.873	−0.04697
Normal-MILD DYS	−0.036	1.000	−0.00175
Normal-MOD DYS	−0.280	1.000	−0.01346
Normal-SEV DYS	−0.132	1.000	−0.00638
Normal-C IN SITU	0.292	1.000	0.01404
Normal-SQ	2.045	0.193	0.09915

**Table A8:** HCC Dunnett Contrasts and effect size.

Hypotheses	*t*-Value	Pr(>|t|)	Effect Size
Normal-LGDLT	−0.021	1.000	−0.00124
Normal-HGDLT	−0.033	1.000	−0.00192
Normal-VEHC	−0.729	0.950	0.04268
Normal-EHC	0.729	0.950	0.04269
Normal-AHC	0.919	0.868	0.05339
Normal-VAHC	0.877	0.890	0.05102

### Appendix A.3. Goodness of Fit to Test Log-Normal Distribution for Pancreatic Cancer and HCC

Gene expression data were tested for a log-normal distribution or a normal distribution. The following results are only for one sample of each stage in the four types of cancer. The other samples render similar results with significant approximations to the tested distributions.

In pancreatic cancer and HCC Kolmogorov-Smirnov test was made to probe the log-normal distribution.

H0: The sample follows a log-normal distribution
Ha: The sample does not follow a log-normal distribution
If *D_n_* < *D_nα_* then the data is a good fit with the log-normal distribution.

In the plots, there is a very good fit with the log normal distribution.

α=0.05 for an n>35; $D_{n,\alpha }=\frac{\sqrt{-0.5\ln\left(\frac{\alpha }{2}\right)}}{\sqrt{n}};$ $D_{n,\alpha }=\frac{1.358}{55.25}=0.024;$ *n* is the number of genes used.

**Table A9:** Kolmogorov-Smirnov test for Pancreatic cancer.

Cancer Stage	Kolmogorov-Smirnov Statistic (Calculated *D_n_* Value)
Normal	0.03062797
IPMA	0.04904261
IOIPMN	0.04815737
IPMC	0.05123668

Since *D_n_* < *D_nα_* we conclude by Kolmogorov-Smirnov that the data does not fit a log-normal distribution, but it is a good aproximation as seen by all plots and the near *D* values.

α=0.05 for an n>35; $D_{n,\alpha }=\frac{\sqrt{-0.5\ln\left(\frac{\alpha }{2}\right)}}{\sqrt{n}};$ $D_{n,\alpha }=\frac{1.358}{24.26}=0.0597$.

**Table A10:** Kolmogorov-Smirnov test for HCC.

Cancer Stage	Kolmogorov-Smirnov Statistic (Calculated *D_n_* Value)
Normal	0.05043655
LGDLT	0.05035987
HGDLT	0.05266466
VEHC	0.04894896
EHC	0.04728102
AHC	0.04770404
VAHC	0.04946804

Since *D_n_* < *D_nα_* we conclude that the data is a good fit with the log-normal distribution.

In melanoma and squamous cell carcinoma of the lung, the Kolmogorov-Smirnov test was made to probe the normal distribution.

H0: The sample follows a normal distribution
Ha: The sample does not follow a normal distribution

If *D_n_* < *D_nα_* it implies that the data is a good fit with the normal distribution.

$$D_{n,\alpha }=\frac{1.358}{44.78}=0.03032$$

**Table A11:** Kolmogorov-Smirnov test for Melanoma.

Cancer stage	Kolmogorov-Smirnov Statistic (Calculated *D_n_* Value)
NS	0.02327633
BN	0.03602832
AN2	0.02572476
INS	0.02299426
VGP	0.02791794
MGP	0.03271799
LN	0.03189103

Since *D_n_* < *D_nα_* we conclude that the data is a good fit with the normal distribution except for BN which is a good approximation.

$$D_{n,\alpha }=\frac{1.358}{29.32}=0.04631$$

**Table A12:** Kolmogorov-Smirnov test for Squamous cell carcinoma.

Cancer Stage	Kolmogorov-Smirnov Statistic (Calculated *D_n_* Value)
NS	0.0451448
HYP	0.04257946
META	0.03065325
MILD DYS	0.03673821
MOD DYS	0.02808452
SEV DYS	0.04279729
C IN SITU	0.03382777
SQ	0.04313791

Since *D_n_* < *D_nα_* we conclude that the data is a good fit with the normal distribution.

## Appendix B. Method to Calculate the Determinant of a Matrix, Tables about the Statistics of Local Networks for Each Cancer, and a Glossary of Terms

### Appendix B.1. Calculation of the Determinant of the Covariance Matrix

A covariance matrix of *X* is a square *k*×*k* matrix whose generic (*i,j*)-th entry is equal to the covariance between *x_i_* and *x_j_* The diagonal entries are equal to the variances of the individual components of *X* (see equation below). Assuming two samples, the 2×2 covariance matrix is given by:

$$k=\left[\begin{array}{cc} Var[X_{1}] & Cov[X_{1},X_{2}]\\ Cov[X_{2},X_{1}] & Var[X_{2}] \end{array}\right]$$

To calculate the determinant of the covariance matrix, we have:

$$|\kappa |=\left[\begin{array}{cc} 1 & 0.3\\ 0.3 & 1 \end{array}\right]$$

|κ|=(1×1)−(0.3×0.3)

|κ|=0.91

where | | denotes the determinant of the matrix.

The following tables summarize some descriptors of the local networks for each group in each cancer.

**Table A13:** Melanoma local networks and size distribution.

Group *	Number of Local Networks	Size Distribution of Local Networks **
1	8	8:2
2	61	61:2
3	258	258:2
4	181	181:2
5	3	5:3
6	30	29:2, 1:4
7	546	201:3, 130: 4, 65:5, 41:6, 30:8, 27: 7, 13:10, 9:9, 9:11, 7:12, 2:13, 2:14, 2;15, 2:16, 2:19, 1:17, 1:20, 1:21, 1:22
8	925	106:3, 100:4, 104:5, 85:6, 73:7, 63:8, 46: 9, 51:10, 34:11, 25:12, 15:13, 18:16, 14:17, 15:18, 11: 19, 8:20, 7:21, 13:22, 12:23, 6:24, 4:25, 5: 26, 4:27, 2:28, 4:29, 4:30, 4:31, 3:32, 7:33, 4:35, 2: 36, 3:37, 4:38, 3:39, 1:40, 4:41, 2:42, 2:43, 2:44, 1:46, 1:48, 2:50, 2:52, 1:55, 1:62, 2:63, 2:35, 1:66, 1:70, 1: 73, 1:75, 1:90, 1:110, 1:118, 1:162, 1:171

* Group count start from the group with the minimum entropy values. ** First number correspond to the number of local networks; second number corresponds to the size of the local networks, e.g., Group 6 has 29 local networks with 2 nodes and 1 local network with 4 nodes.

**Table A14:** HCC local networks and size distribution.

Group *	Number of Local Networks	Size Distribution of Local Networks **
1	9	9:2
2	68	68:2
3	102	11:3, 91:2
4	91	91:2
5	42	31:4, 11:3
6	43	1:5, 42:4
7	47	47:5
8	31	31:6
9	47	16:7, 31:6
10	34	4:9, 16:8, 14:7
11	106	1:83, 1:63, 1:55, 1:54, 1:50, 1:43, 1:40, 1:38, 1:32, 1:30, 1:29, 1:26, 2:25, 3:23, 1:22, 2:21, 3:20, 3:19, 4:18, 7:17, 5:16, 3:15, 6:14, 4:13, 8:12, 7:11, 12:10, 8:9, 16:8

* Group counts start from the group with the minimum entropy value. ** First number corresponds to the number of local networks; second number corresponds to the size of the local networks, e.g., Group 1 has 9 local networks with 2 nodes.

**Table A15:** Pancreatic local networks and size distribution.

Group *	Number of Local Networks	Size Distribution of Local Networks **
1	87	87:2
2	341	341:2
3	26	1:3, 25:2
4	17	17:3
5	267	267:3
6	10	10:2
7	22	2:5, 20:4
8	1170	203:4, 150:5, 138:6, 104:7, 82:8, 72:9, 63:10, 45:11, 38:12, 33:13, 32:14, 19:15, 17:19, 13:20, 12:17, 9:26, 9:25, 9:21, 9:18, 8:29, 8:24, 8:23, 6:22, 5:33, 4:30, 4:28, 4:27, 3:51, 3:47, 3:43, 3:36, 3:32, 2:79, 2:62, 2:55, 2:53, 2:49, 2:41, 2:37, 2:35, 2:34, 1:168, 1:128, 1:89, 1:83, 1:78, 1:77, 1:69, 1:66, 1: 63, 1:61, 1:54, 1:42, 1:39, 1:38, 1:31
9	5	1:40, 1:19, 1:10, 1:7, 1:4

* Group counts start from the group with the minimum entropy value. ** First number corresponds to the number of local networks; second number corresponds to the size of the local networks, e.g., Group 3 has 1 local network with 3 nodes and 25 local networks with 2 nodes.

**Table A16:** Squamous cell carcinoma local networks and size distribution.

Group *	Number of Local Networks	Size Distribution of Local Networks **
1	16	16:2
2	117	2:3, 115:2
3	174	16:3, 158:2
4	143	126:3, 17:2
5	77	36:4, 41:3
6	67	1:5, 66:4
7	49	48:5, 1:4
8	33	24:6, 9:5
9	33	2:7, 26:6
10	32	31:7, 1:6
11	29	11: 8, 2:7
12	13	1: 9, 12:8
13	11	11:9
14	11	11:10
15	7	7:11
16	4	1:11, 3:12
17	3	3:12
18	28	1: 24, 2:23, 1:21, 1:18, 5:17, 1:16, 4:15, 6:14, 7:13
19	12	1: 57, 1:56, 1:37, 1:36, 1:35, 1:33, 1:29, 2:24, 1:22, 1:18, 1:16, 1:14

* Group count start from the group with the minimum entropy values. ** First number corresponds to the number of local networks. Second number corresponds to size of the local networks, e.g., Group 12 has 1 local network with 9 nodes and 12 local networks with 8 nodes.

### Appendix B.2. Glossary

Sample—It is the gene expression levels coming from one individual.
Network—All the nodes from the APID PPI data that represents the selected genes from the differential expression analysis for each stage in each type of cancer.
Subnetwork—A group of local networks binned together based on similarity of Multivariate Normal entropy in the normal group.
Local network—It is one node of the network plus its immediate neighbors based on the APID PPI data.
