# Supplementary material for: Multivariate Entropy Characterizes the Gene Expression and Protein-Protein Networks in Four Types of Cancer
Source: Entropy (Basel). 2018 Feb 28;20(3):154. doi: 10.3390/e20030154 (PMC7844632; doi:10.3390/e20030154)
Supplement: Supplementary file 1 [file entropy-20-00154-s001.pdf]

Supplementary Material
Multivariate entropy characterizes the gene expression and protein-protein networks in four types of cancer.

Angel Juarez Flores, Marco V. José

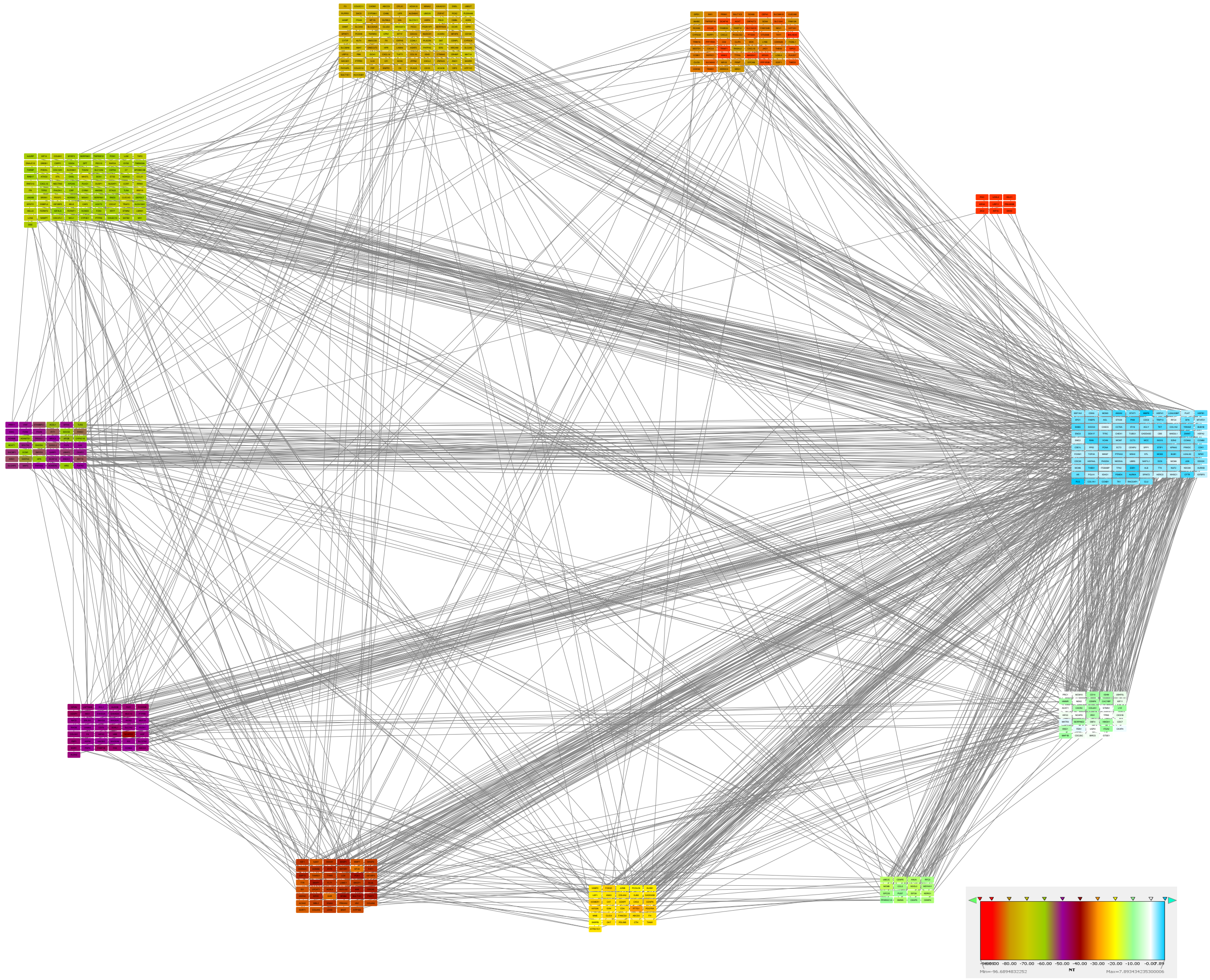

Figure S1. Local network entropy from control PPIs network of HCC carcinogenic process. The multivariate entropy for the local networks of each node and colors assigned to each group are shown.

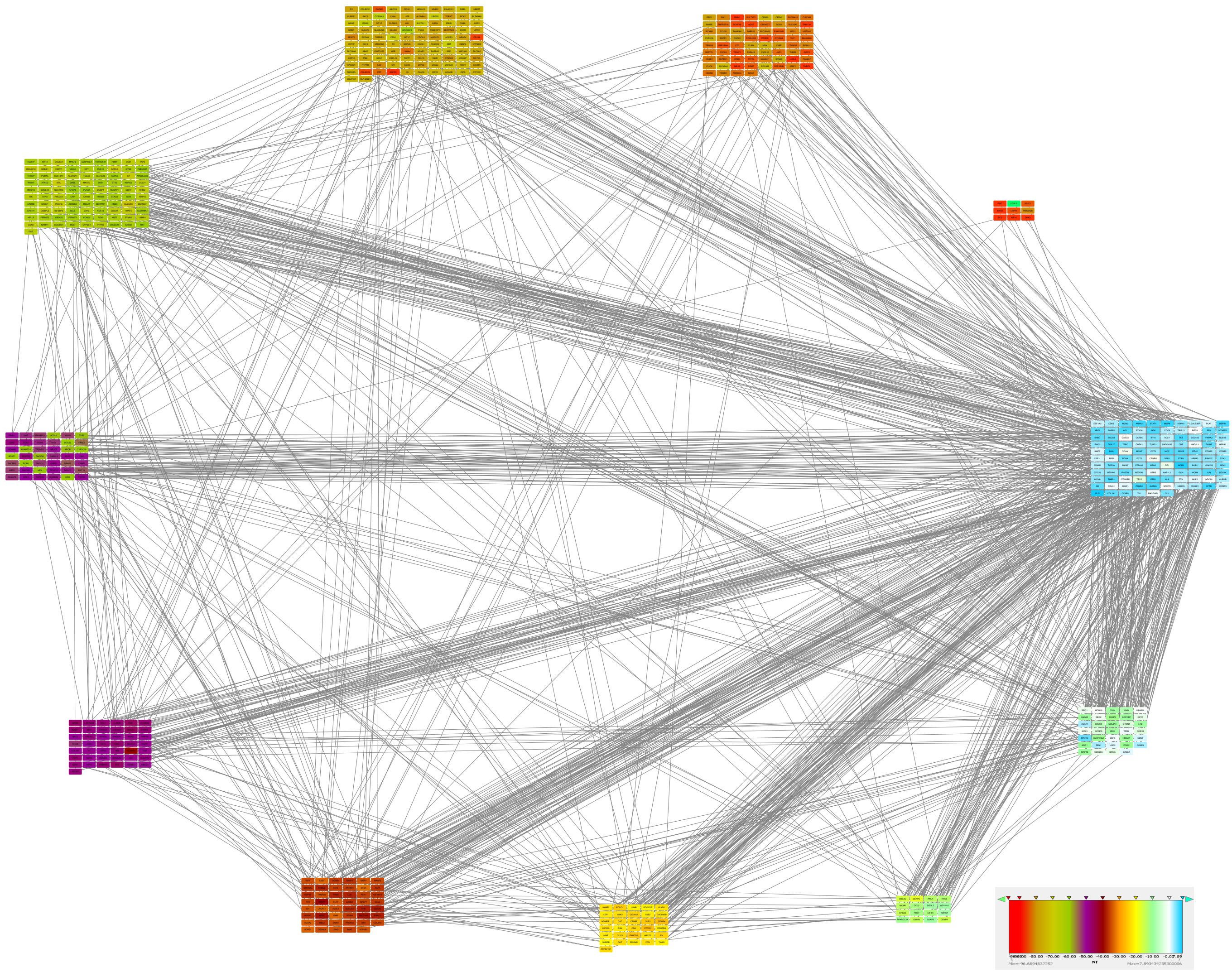

Figure S2. Local network entropy from LGDLT PPIs network of HCC carcinogenic process. The multivariate entropy for the local networks of each node and colors assigned to each group are shown.

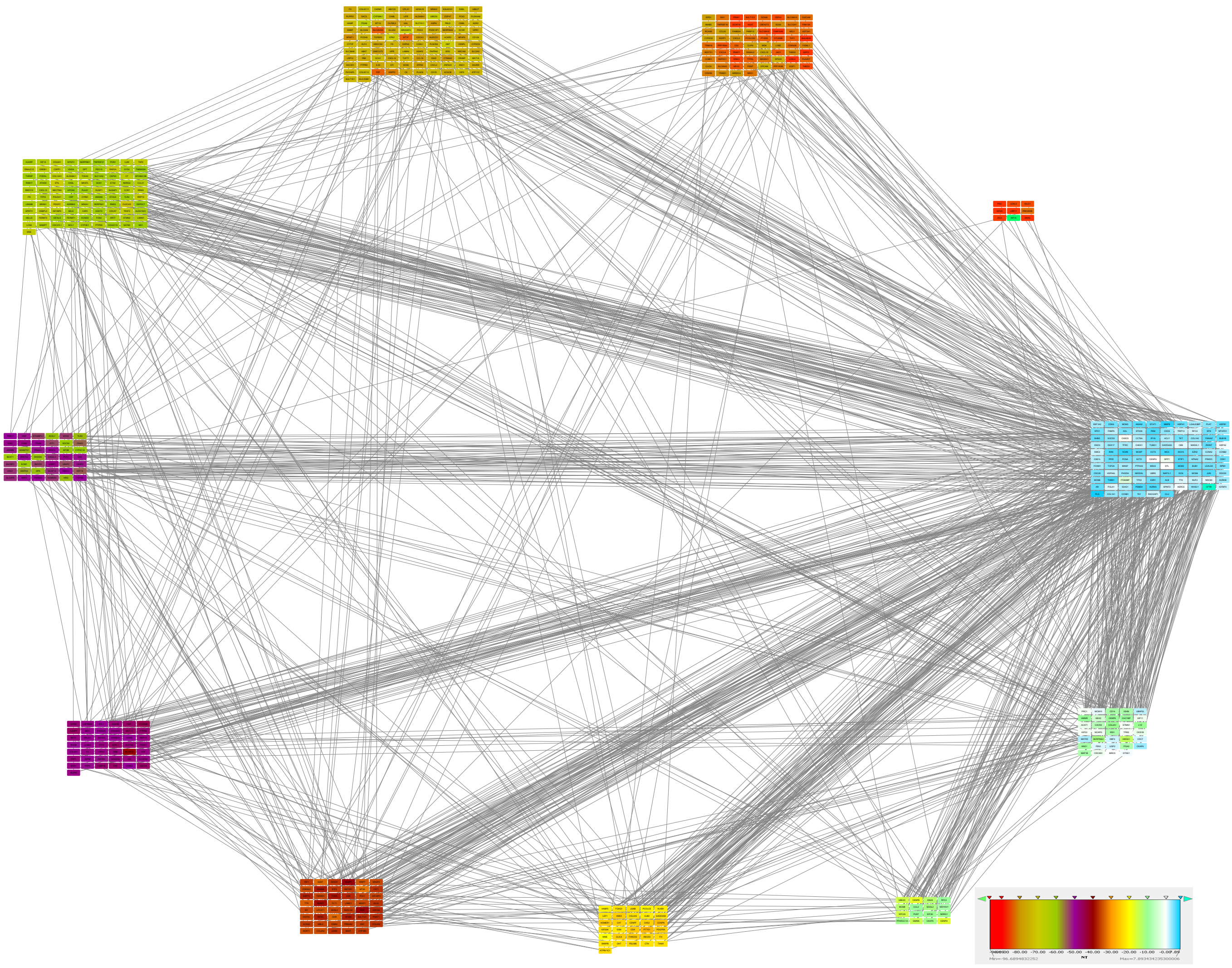

Figure S3. Local network entropy from HGDLT PPIs network of HCC carcinogenic process. The multivariate entropy for the local networks of each node and colors assigned to each group are shown.

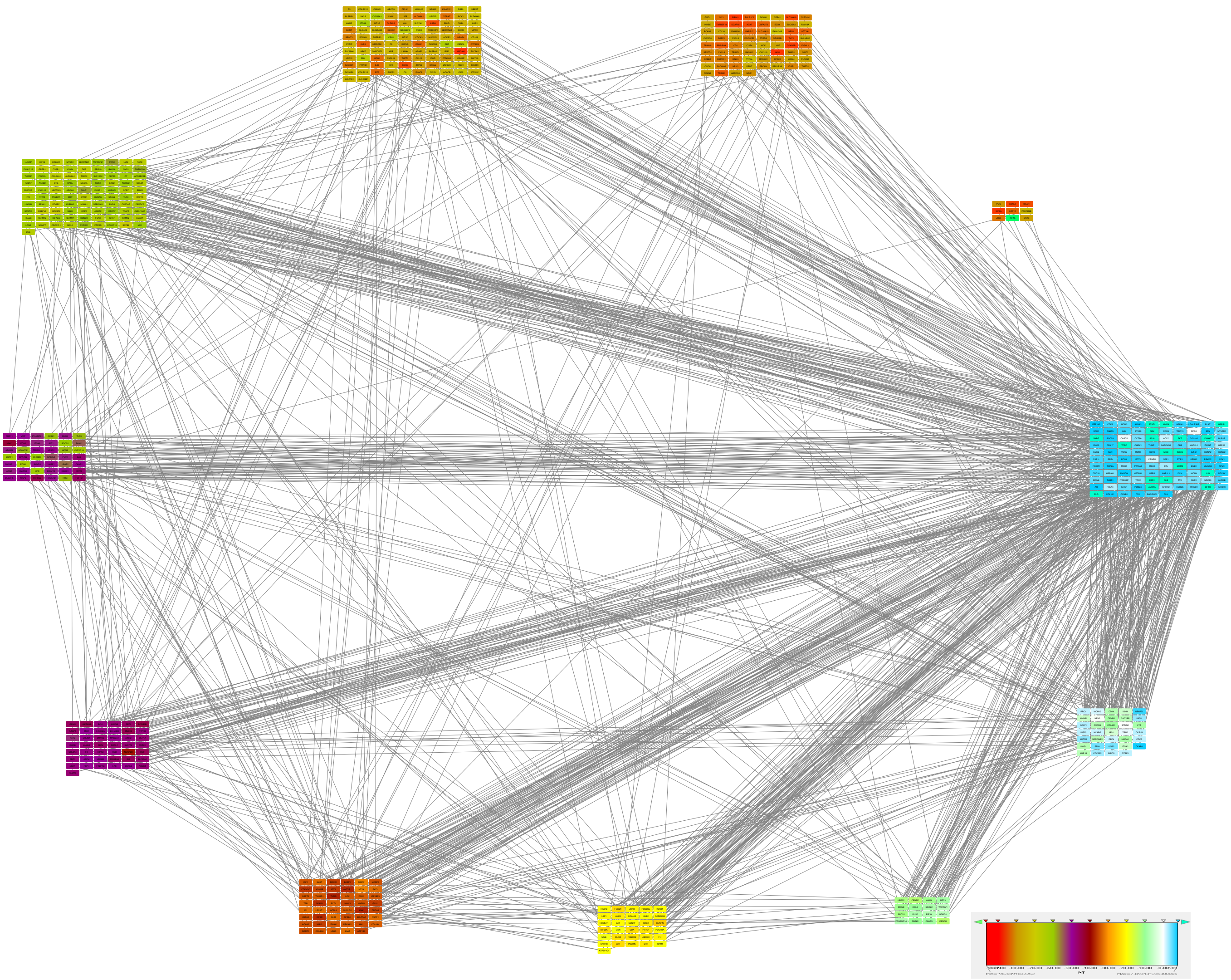

Figure S4. Local network entropy from VEHC PPIs network of HCC carcinogenic process. The multivariate entropy for the local networks of each node and colors assigned to each group are shown.

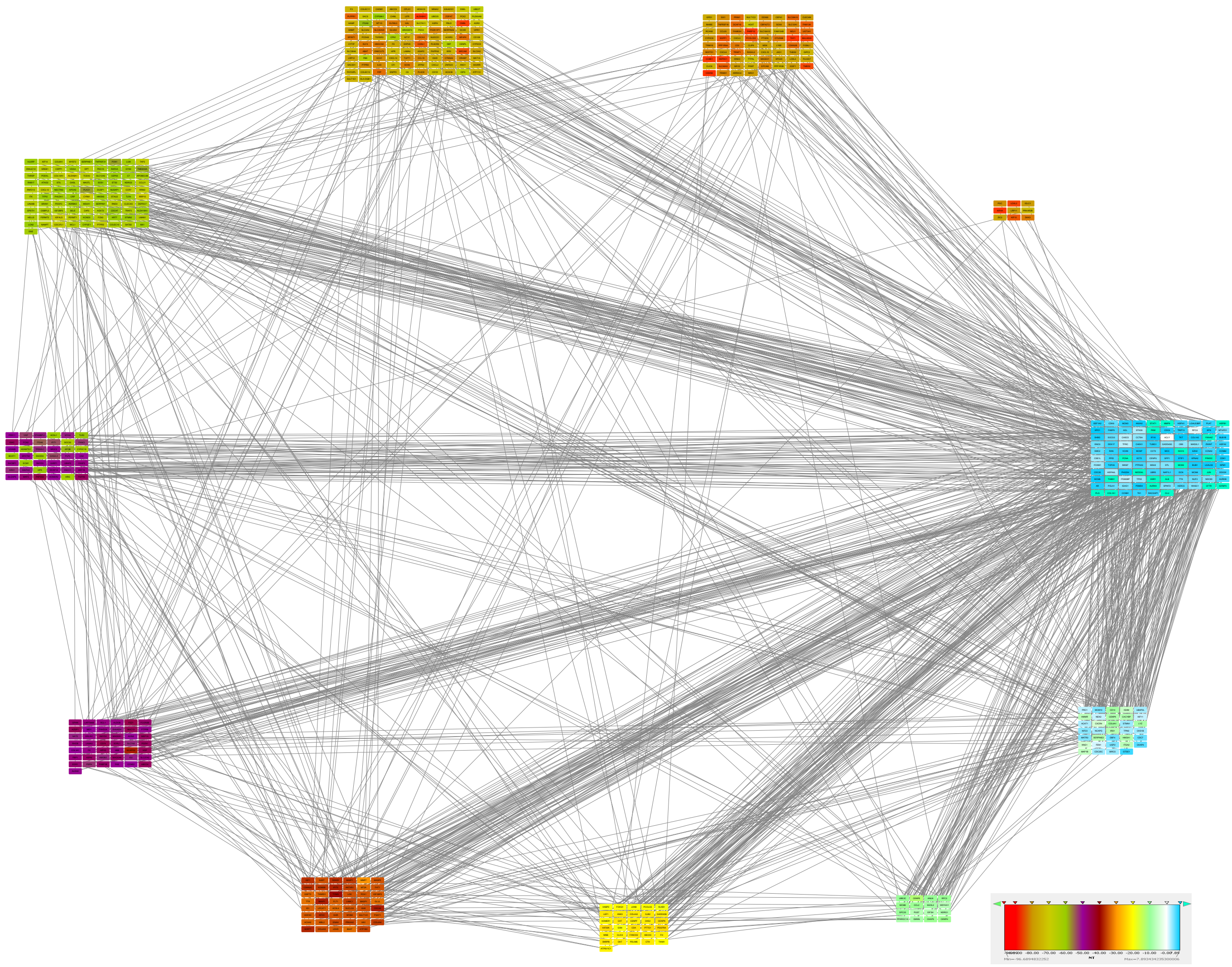

Figure S5. Local network entropy from EHC PPIs network of HCC carcinogenic process. The multivariate entropy for the local networks of each node and colors assigned to each group are shown.

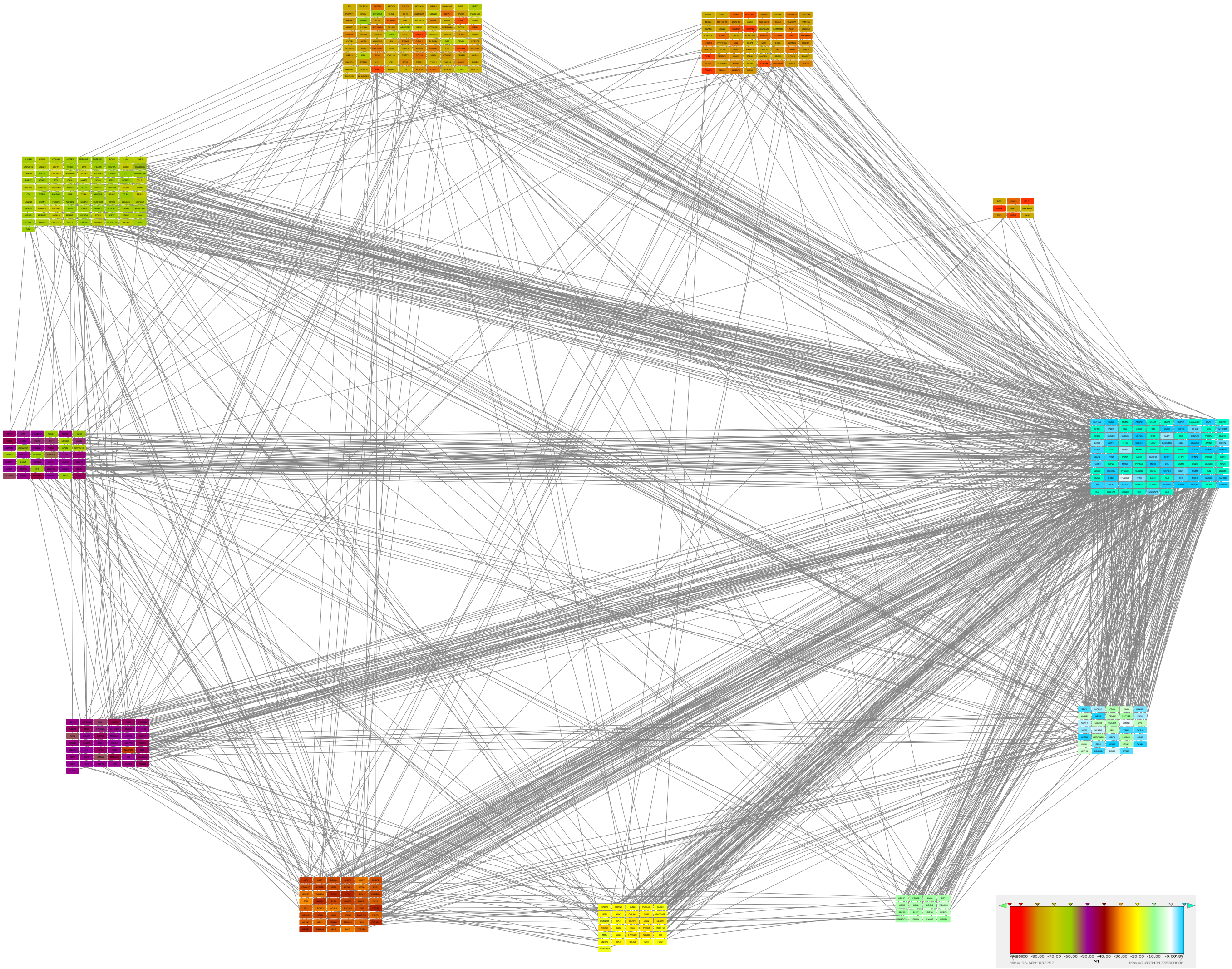

Figure S6. Local network entropy from AHC PPIs network of HCC carcinogenic process. The multivariate entropy for the local networks of each node and colors assigned to each group are shown.

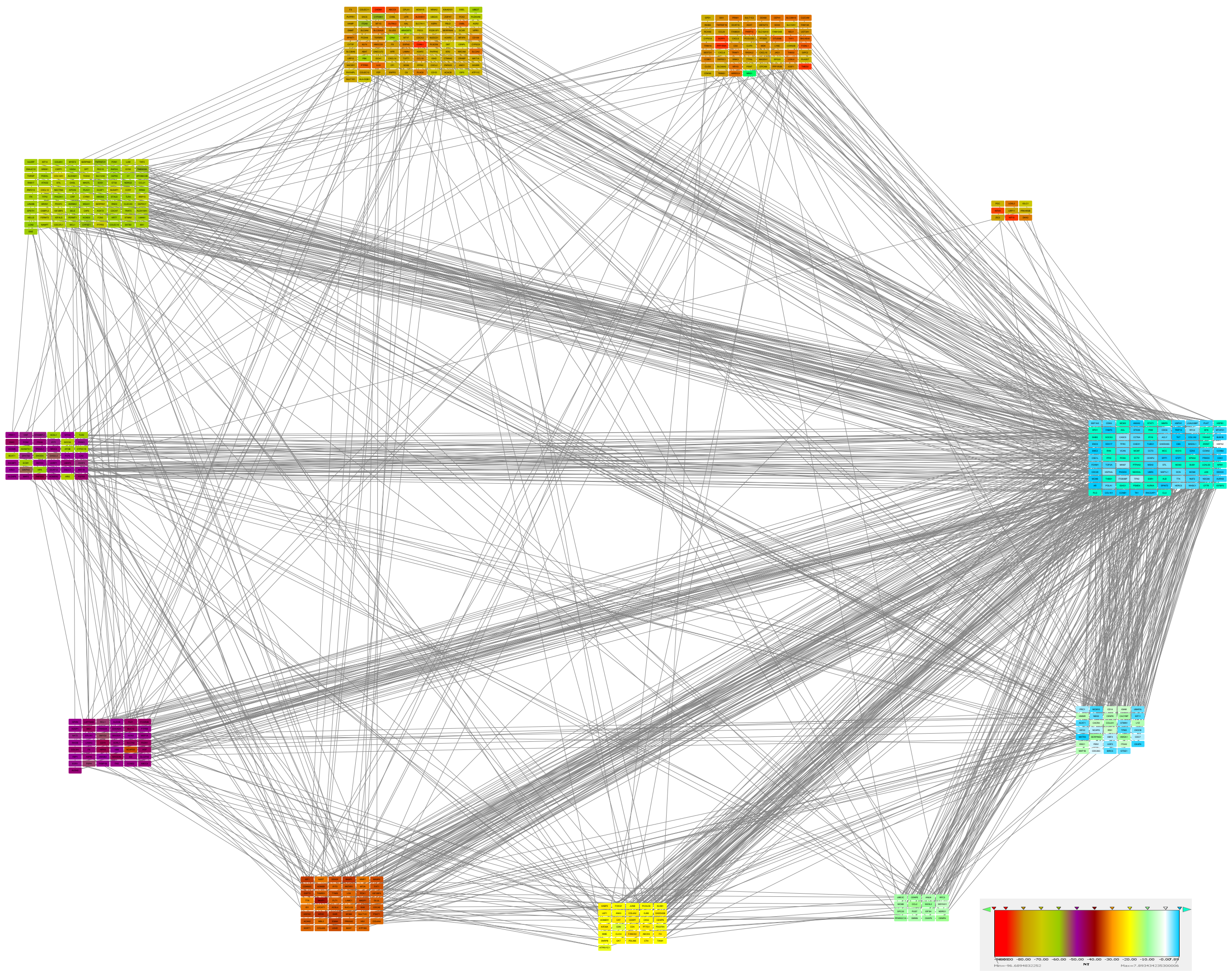

Figure S7. Local network entropy from VAHC PPIs network of HCC carcinogenic process. The multivariate entropy for the local networks of each node and colors assigned to each group are shown.

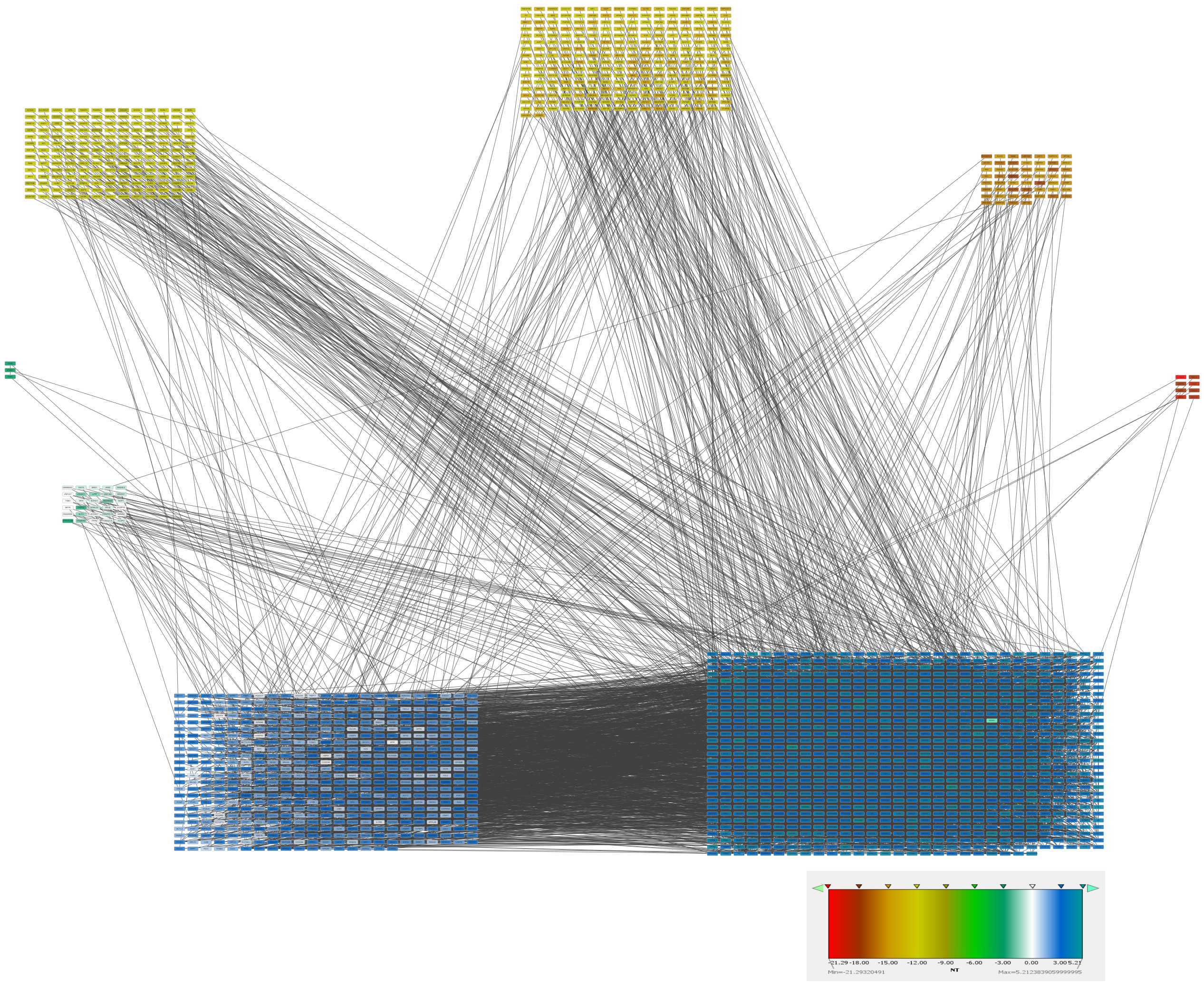

Figure S8. Local network entropy from control PPIs network of Melanoma carcinogenic process. The multivariate entropy for the local networks of each node and colors assigned to each group are shown.

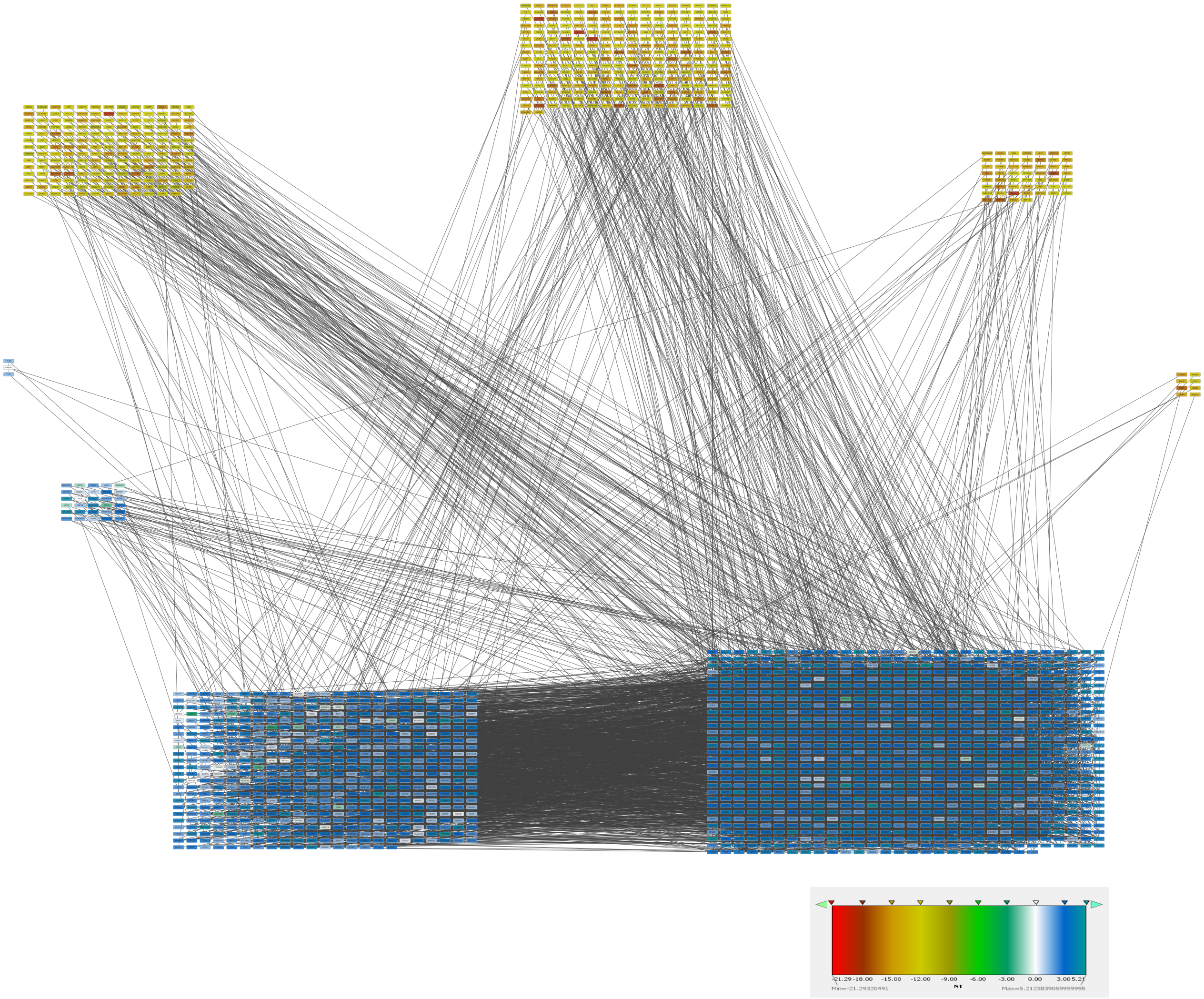

Figure S9. Local network entropy from BN PPIs network of Melanoma carcinogenic process. The multivariate entropy for the local networks of each node and colors assigned to each group are shown.

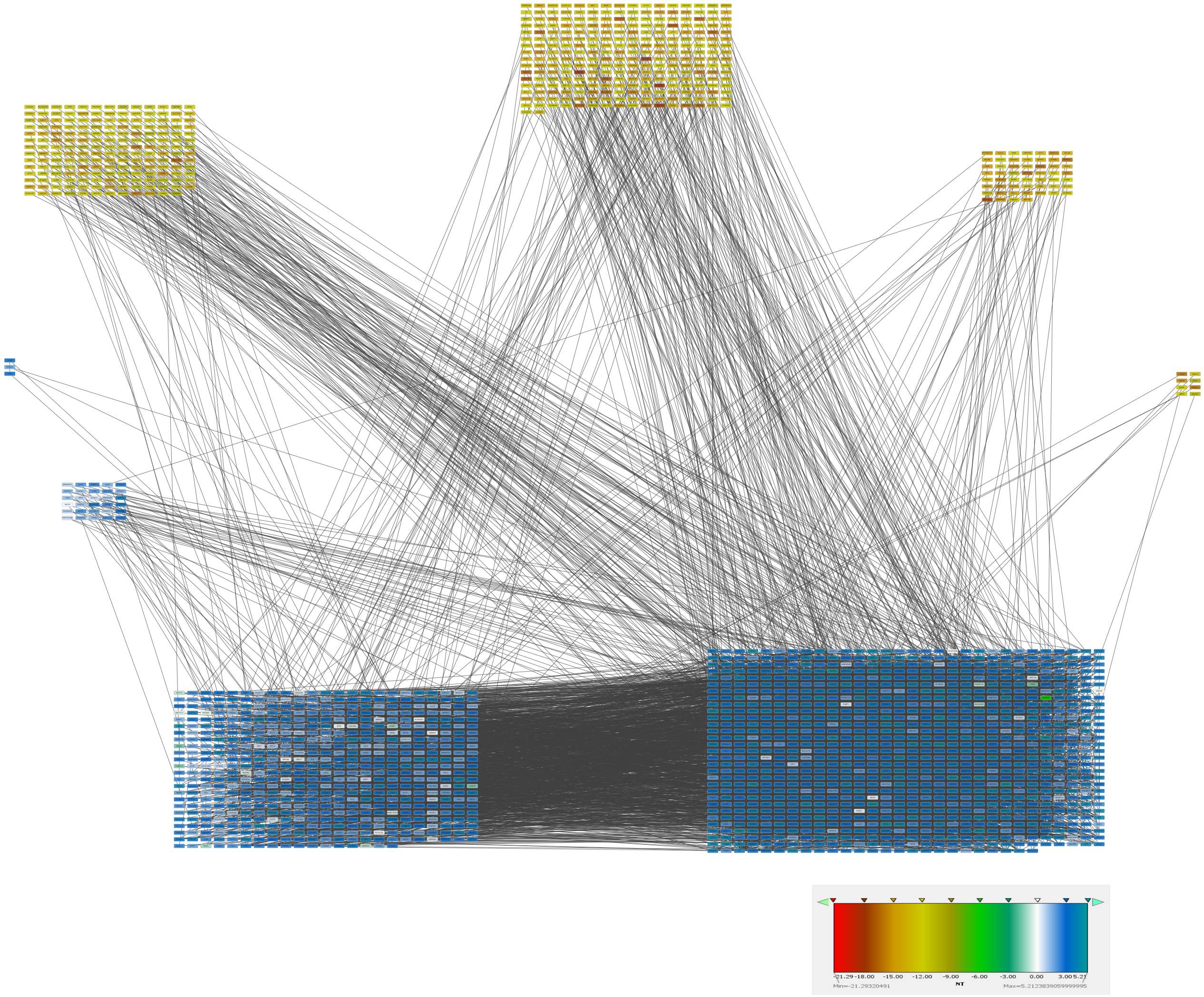

Figure S10. Local network entropy from AN2 PPIs network of Melanoma carcinogenic process. The multivariate entropy for the local networks of each node and colors assigned to each group are shown.

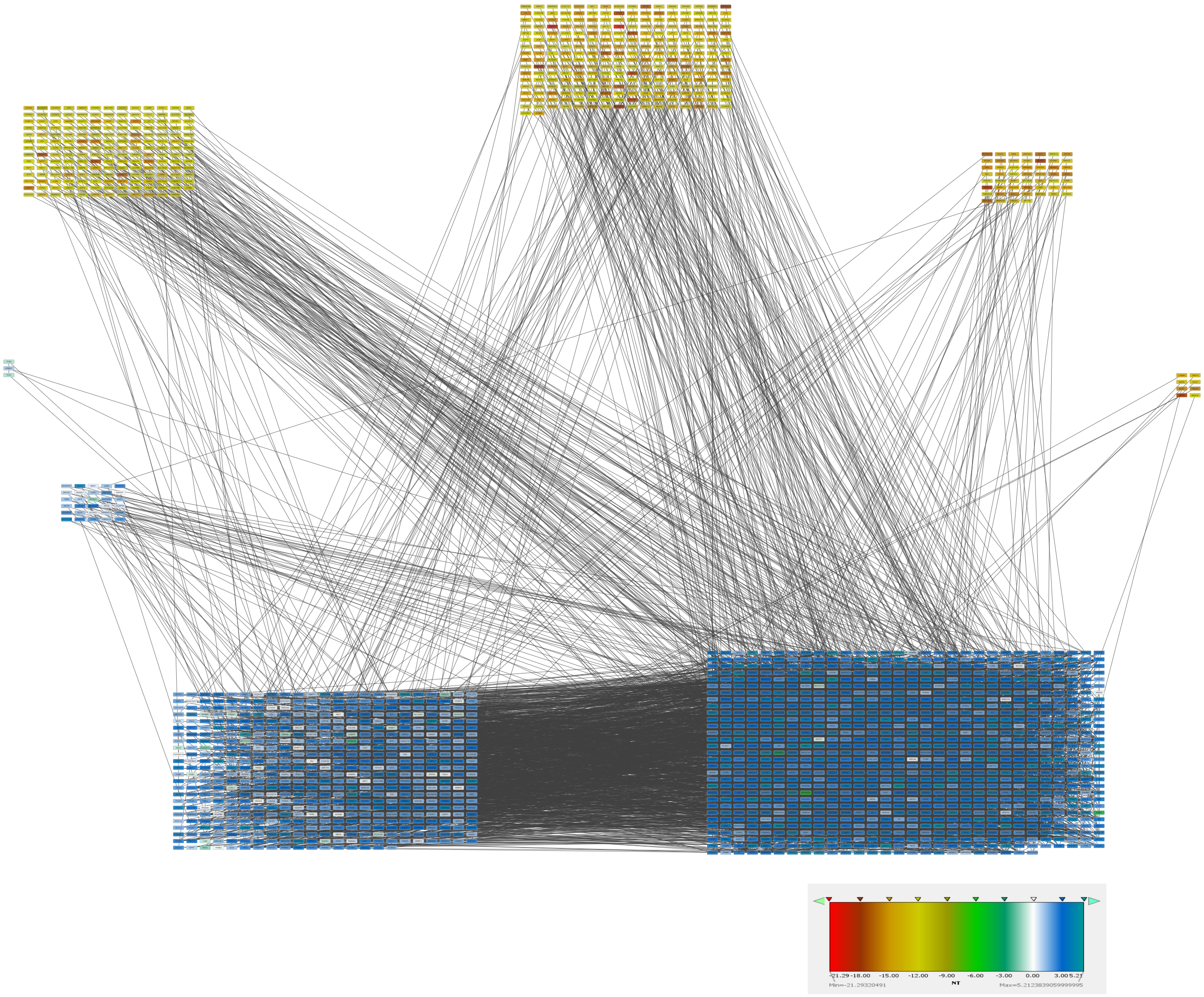

Figure S11. Local network entropy from INS PPIs network of Melanoma carcinogenic process. The multivariate entropy for the local networks of each node and colors assigned to each group are shown.

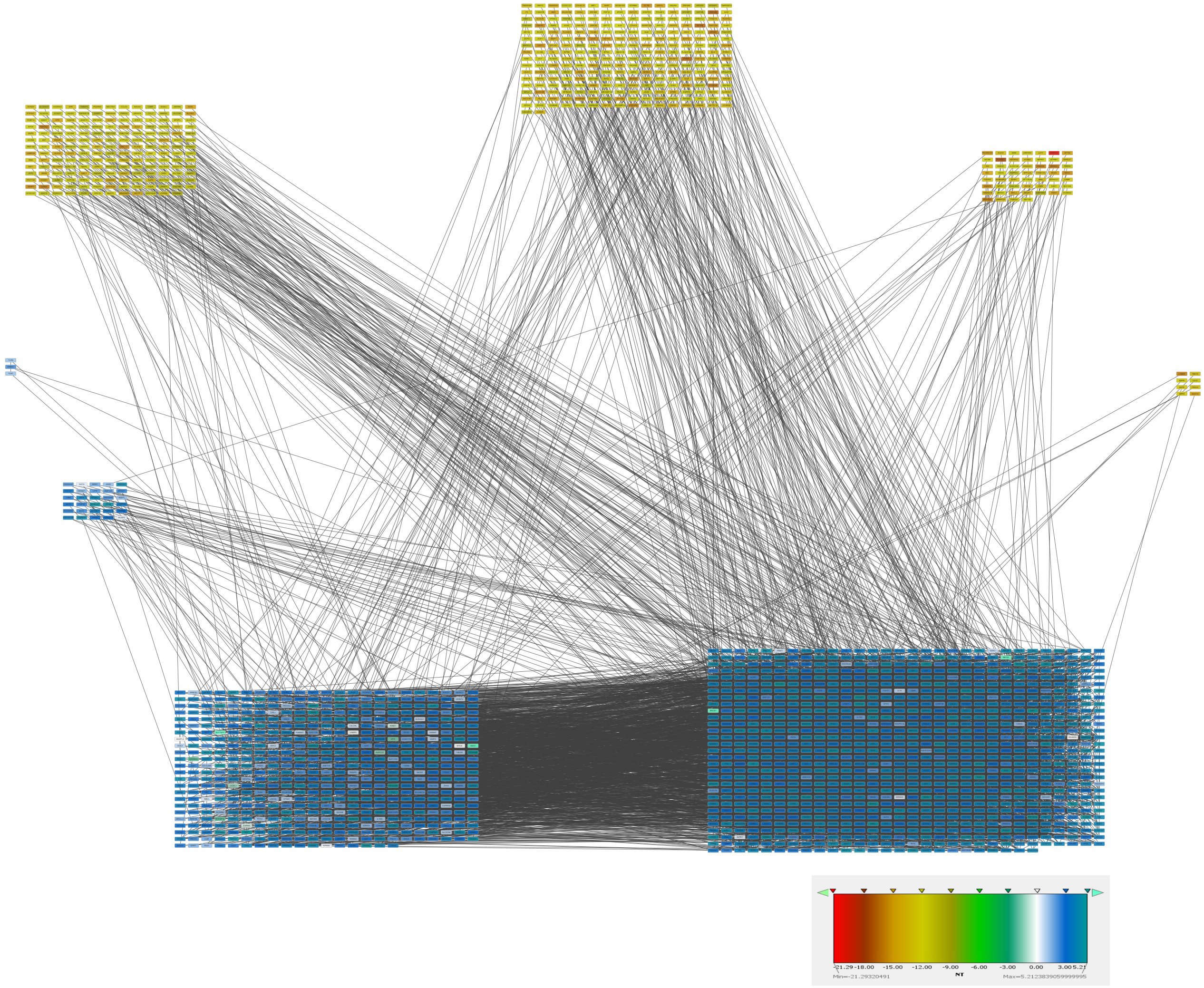

Figure S12. Local network entropy from VGP PPIs network of Melanoma carcinogenic process. The multivariate entropy for the local networks of each node and colors assigned to each group are shown.

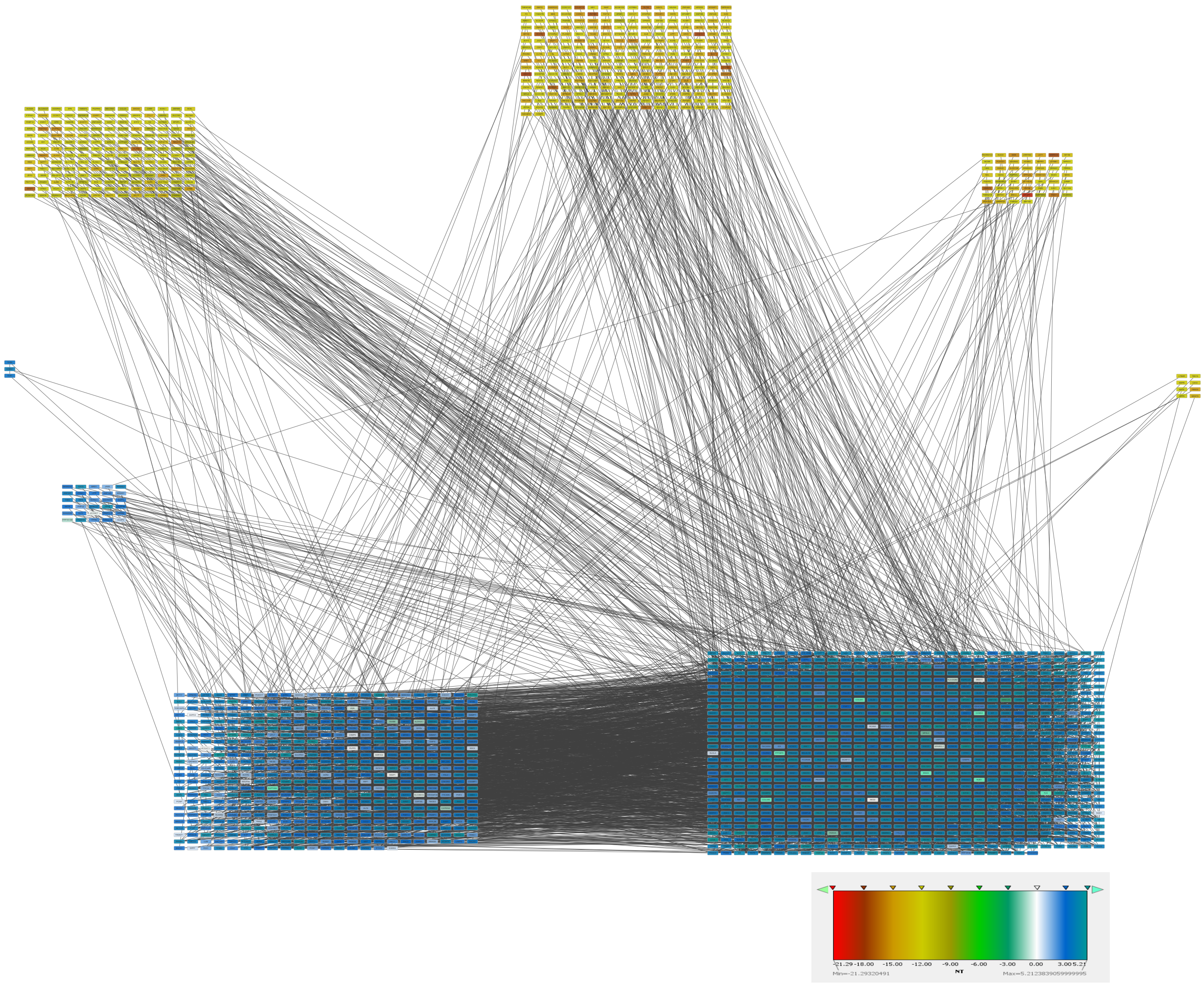

Figure S13. Local network entropy from MGP PPIs network of Melanoma carcinogenic process. The multivariate entropy for the local networks of each node and colors assigned to each group are shown.

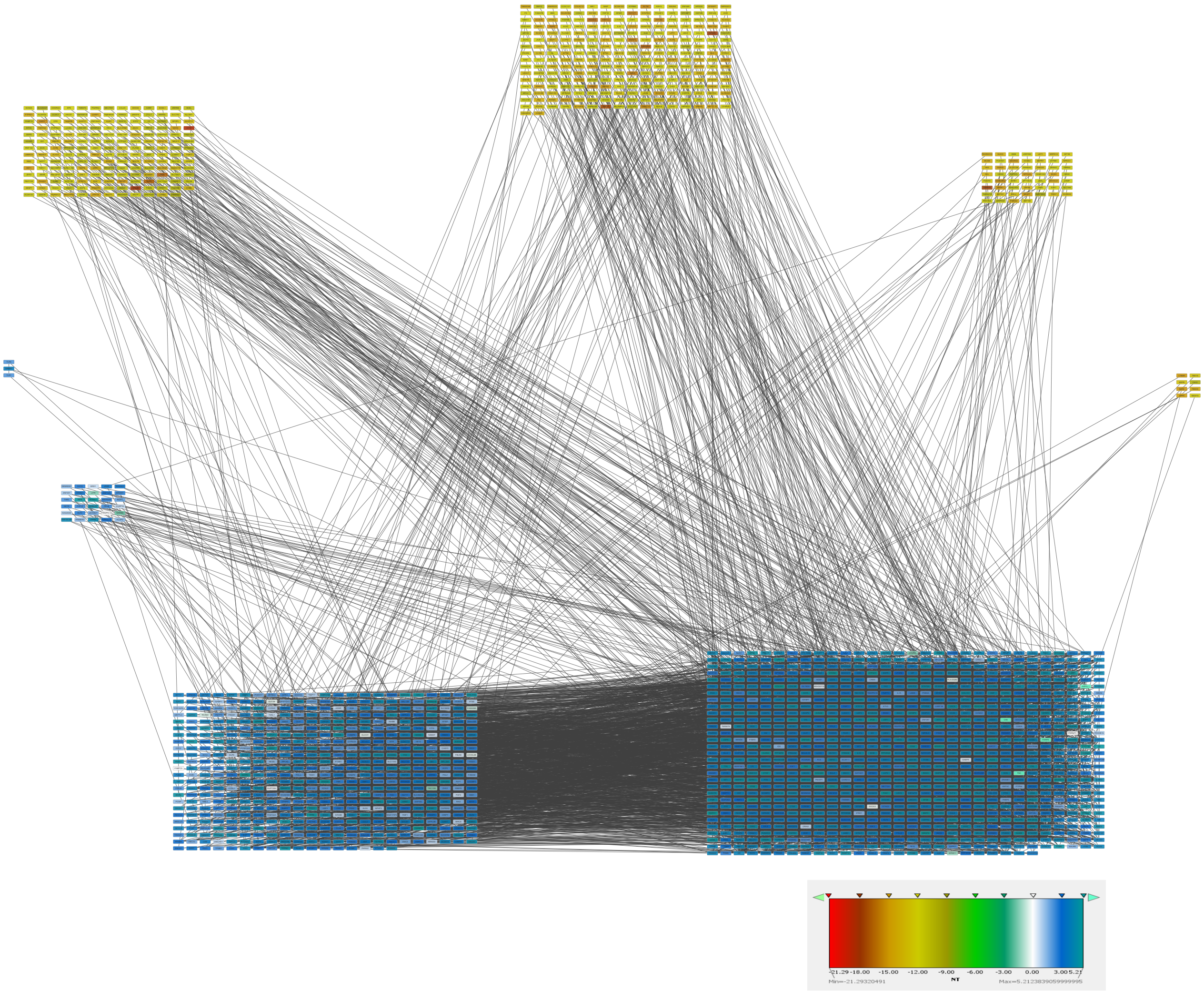

Figure S14. Local network entropy from LN PPIs network of Melanoma carcinogenic process. The multivariate entropy for the local networks of each node and colors assigned to each group are shown.

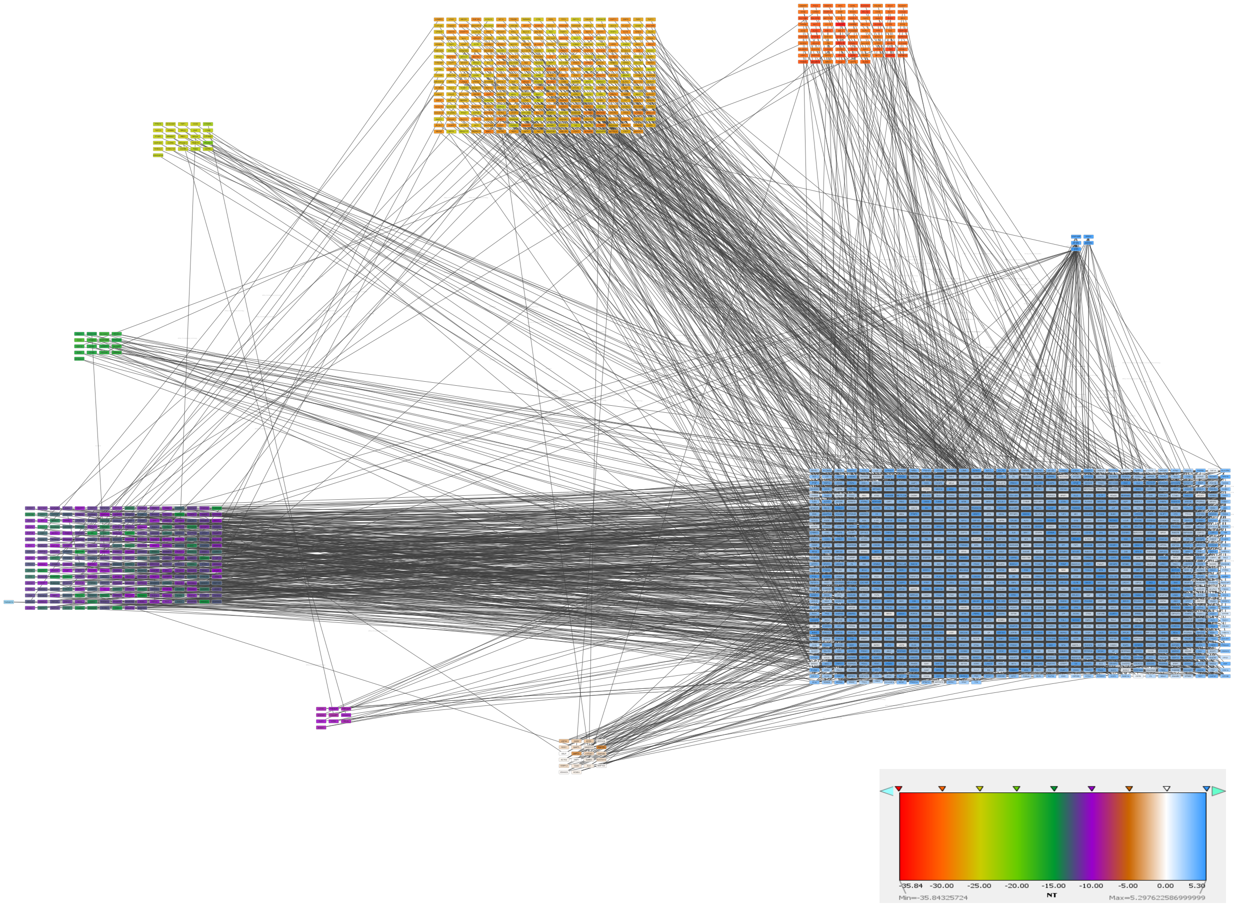

Figure S15. Local network entropy from control PPIs network of Pancreatic cancer carcinogenic process. The multivariate entropy for the local networks of each node and colors assigned to each group are shown.

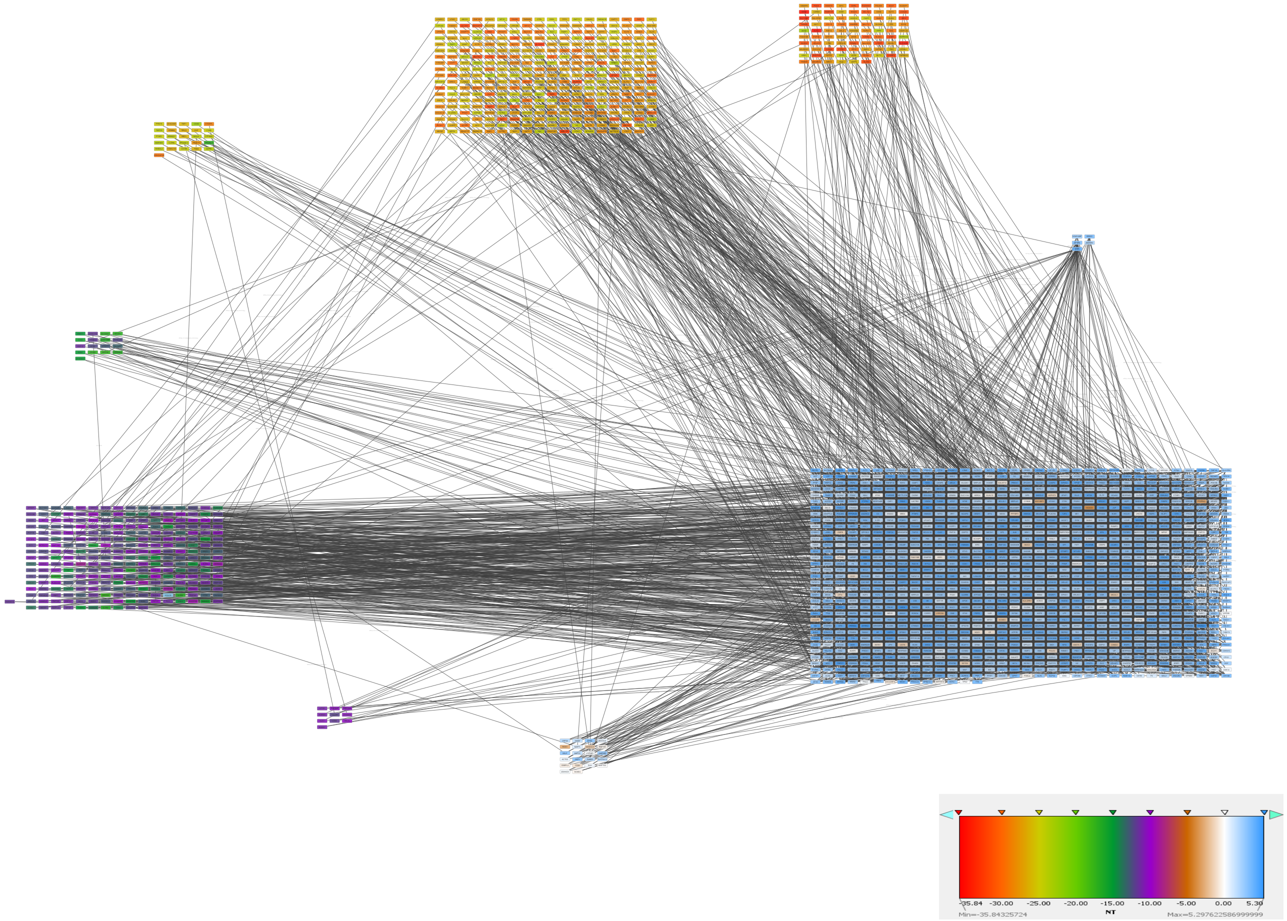

Figure S16. Local network entropy from IPMA PPIs network of Pancreatic cancer carcinogenic process. The multivariate entropy for the local networks of each node and colors assigned to each group are shown.

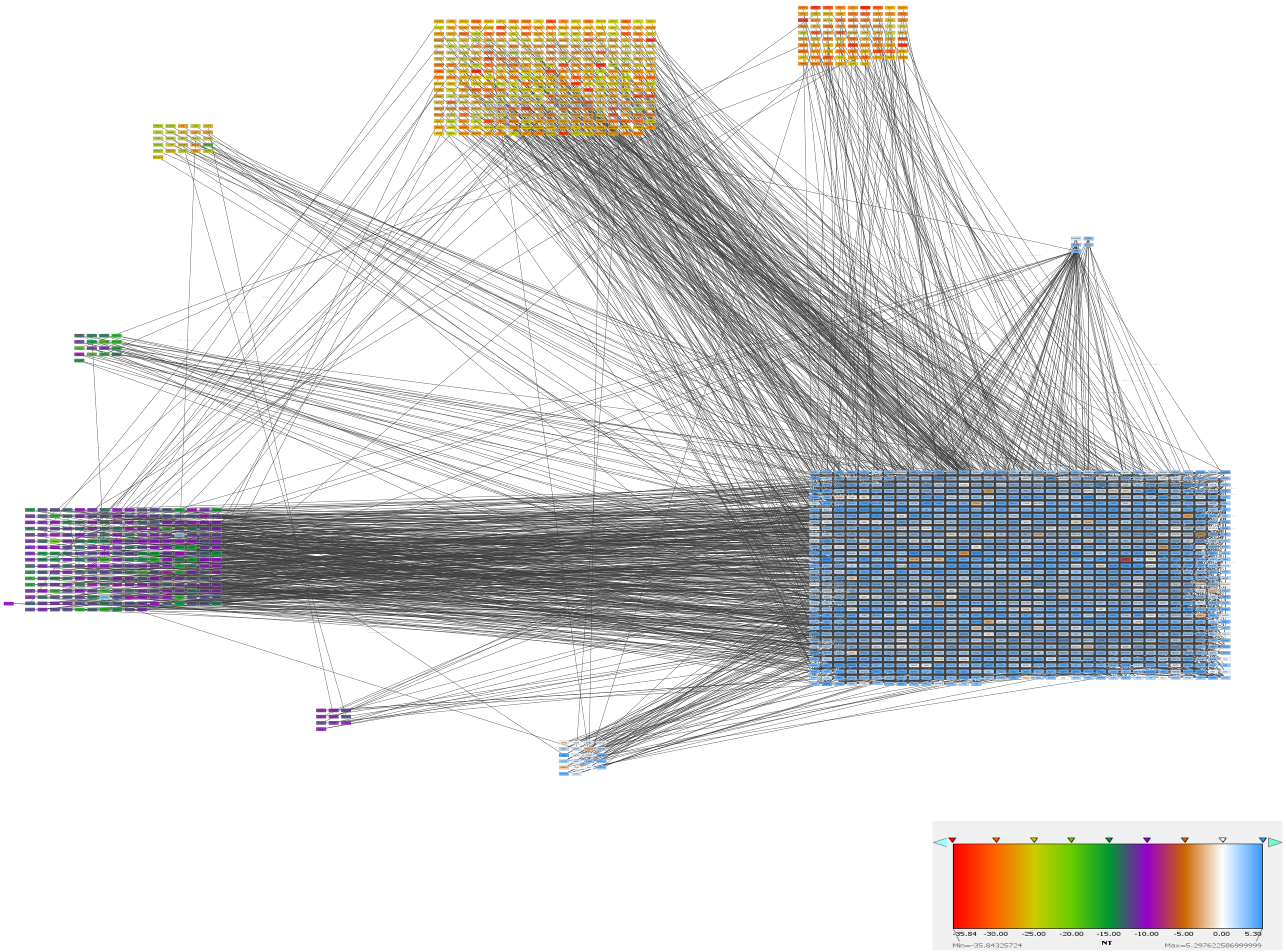

Figure S17. Local network entropy from IOIPMN PPIs network of Pancreatic cancer carcinogenic process. The multivariate entropy for the local networks of each node and colors assigned to each group are shown.

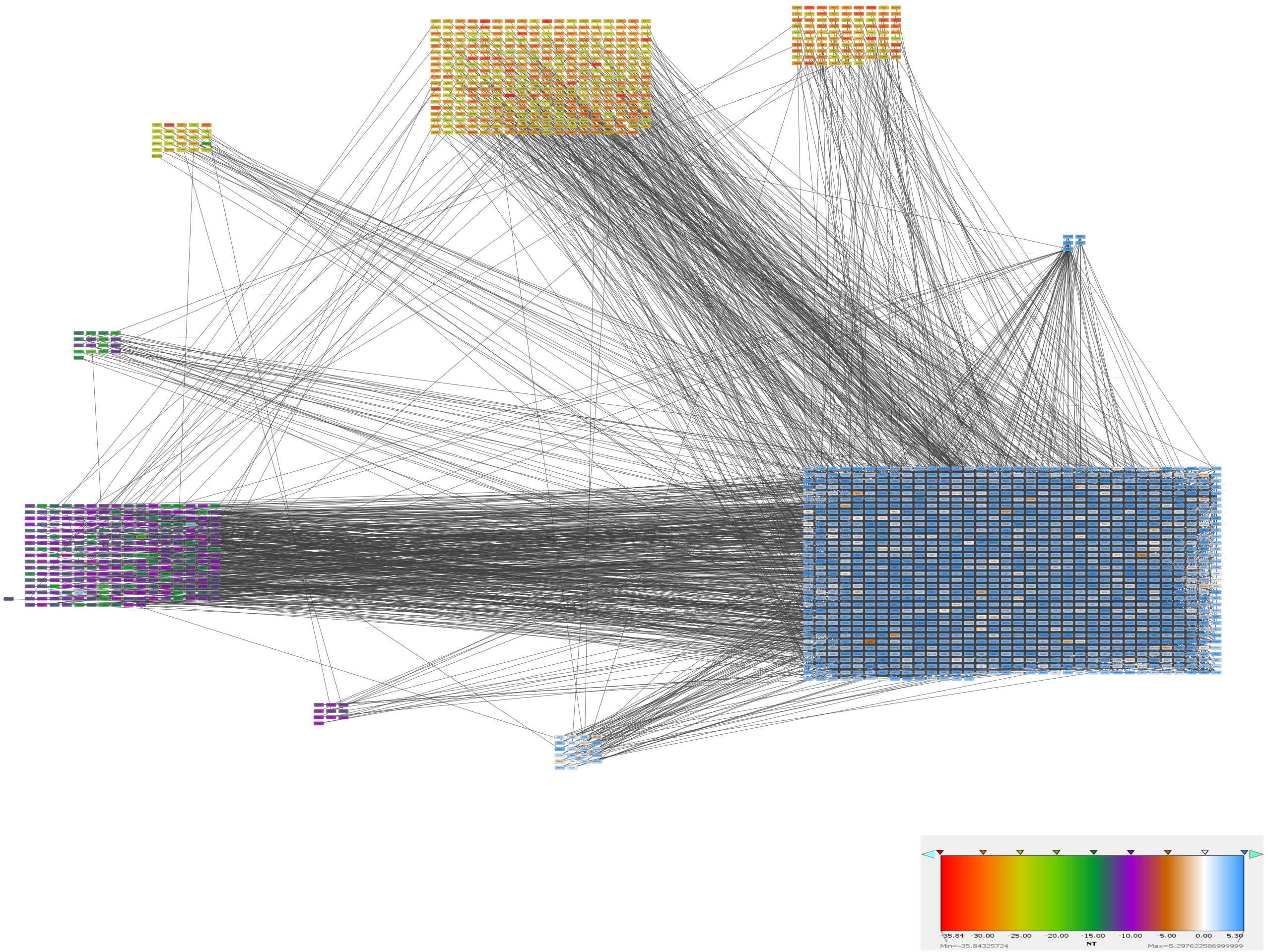

Figure S18. Local network entropy from IPMC PPIs network of Pancreatic cancer carcinogenic process. The multivariate entropy for the local networks of each node and colors assigned to each group are shown.

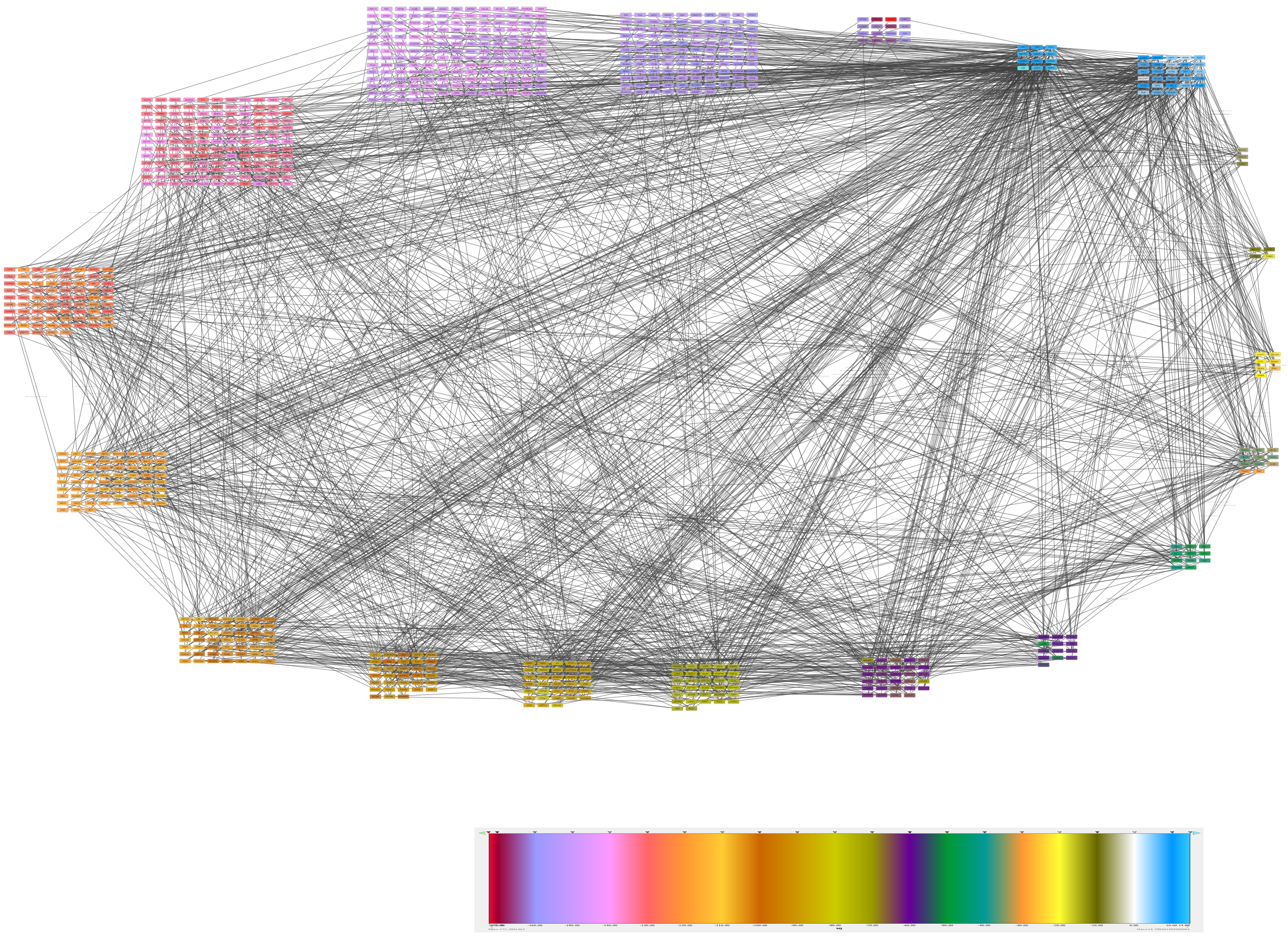

Figure S19. Local network entropy from control PPIs network of Squamous cell carcinoma of the lung carcinogenic process. The multivariate entropy for the local networks of each node and colors assigned to each group are shown.

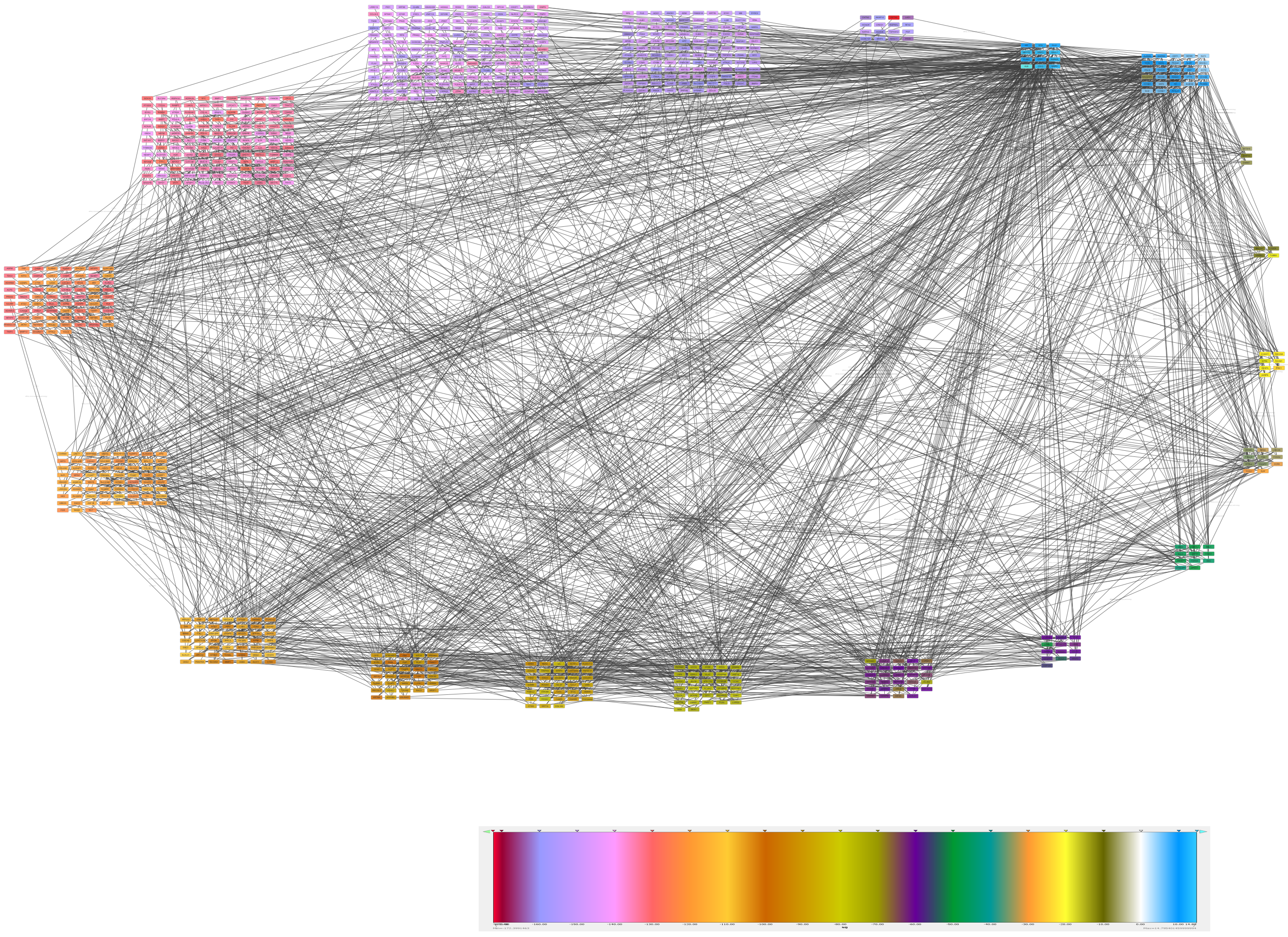

Figure S20. Local network entropy from HYP PPIs network of Squamous cell carcinoma of the lung carcinogenic process. The multivariate entropy for the local networks of each node and colors assigned to each group are shown.

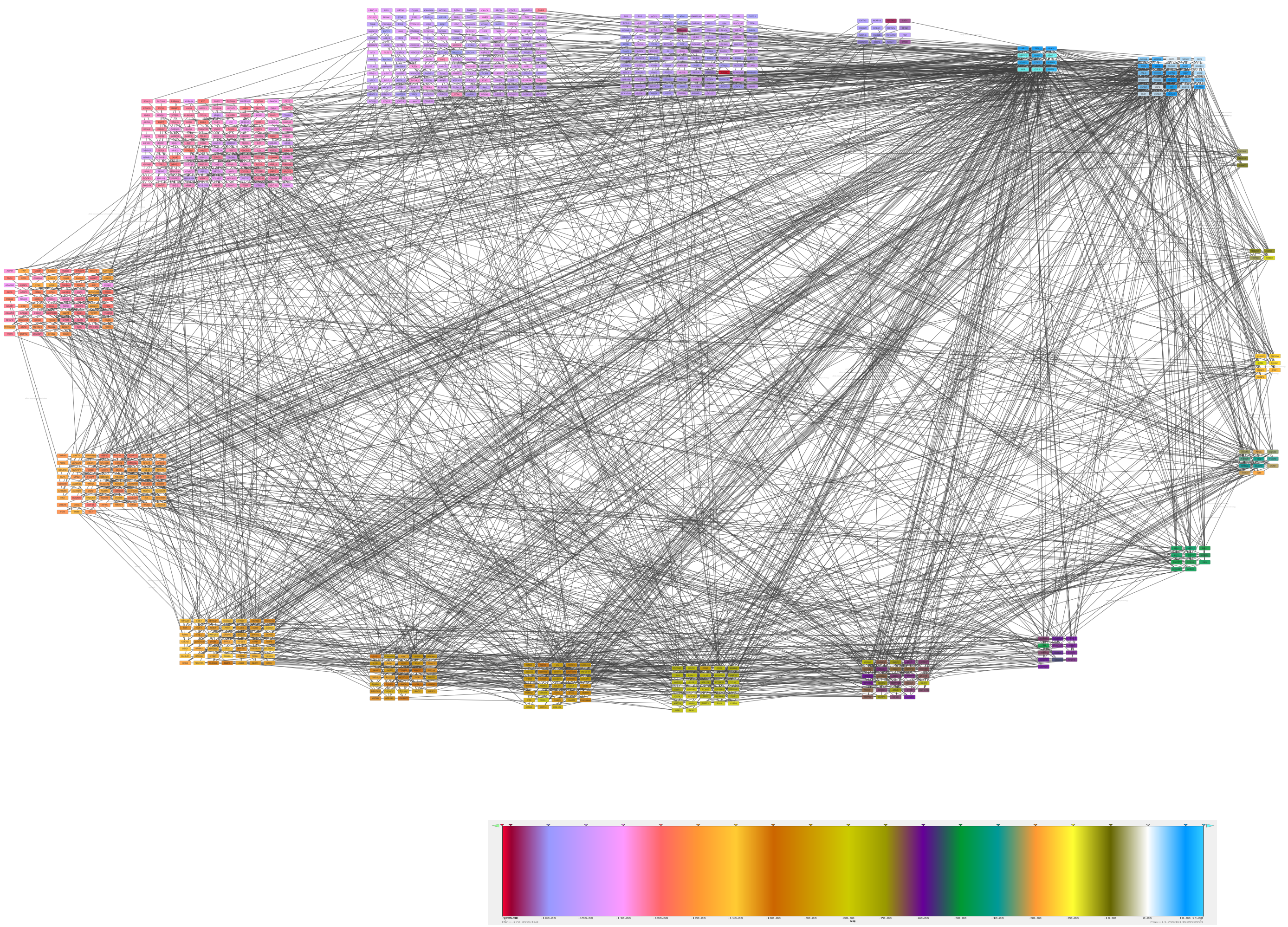

Figure S21. Local network entropy from META PPIs network of Squamous cell carcinoma of the lung carcinogenic process. The multivariate entropy for the local networks of each node and colors assigned to each group are shown.

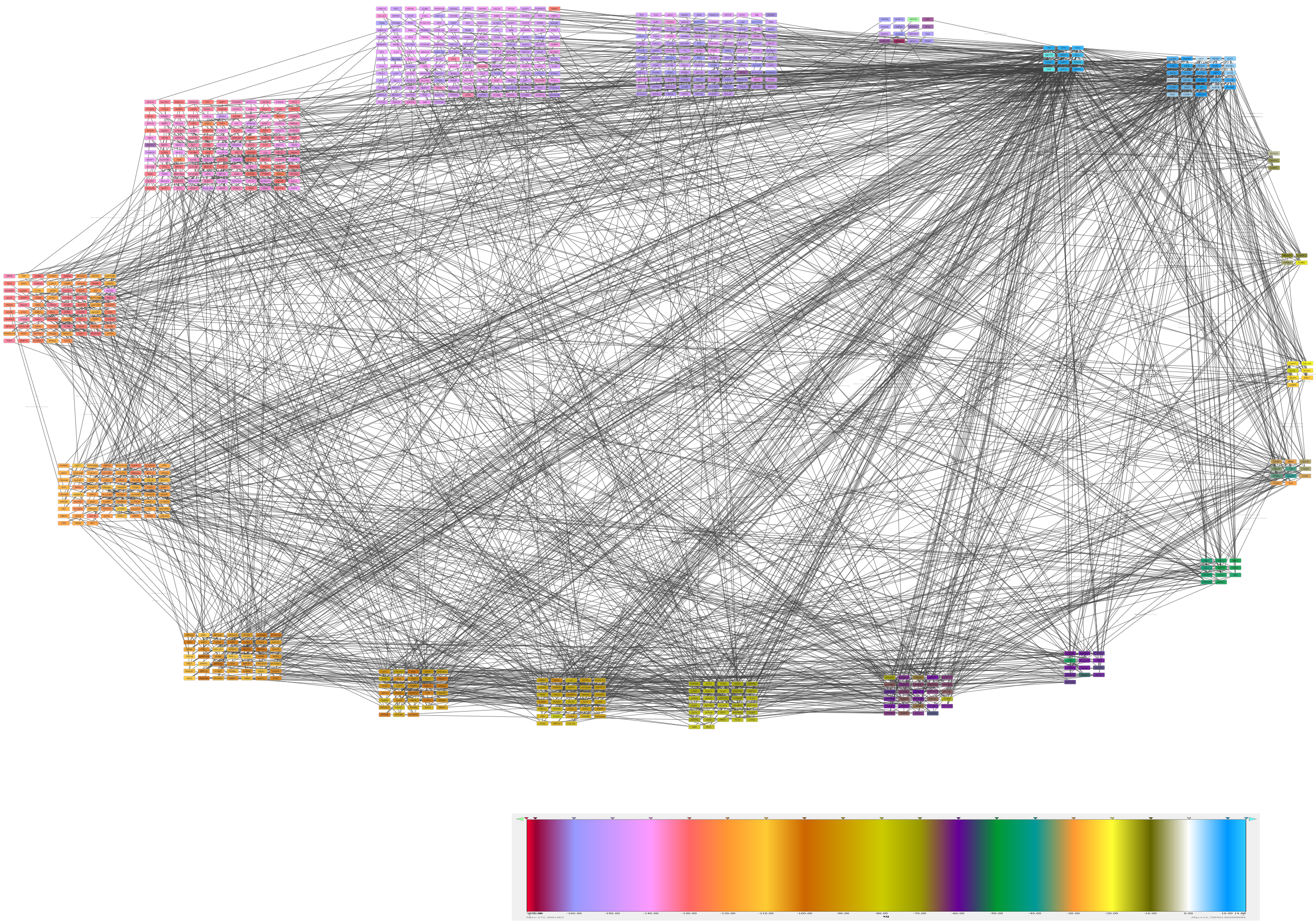

Figure S22. Local network entropy from MILD DYS PPIs network of Squamous cell carcinoma of the lung carcinogenic process. The multivariate entropy for the local networks of each node and colors assigned to each group are shown.

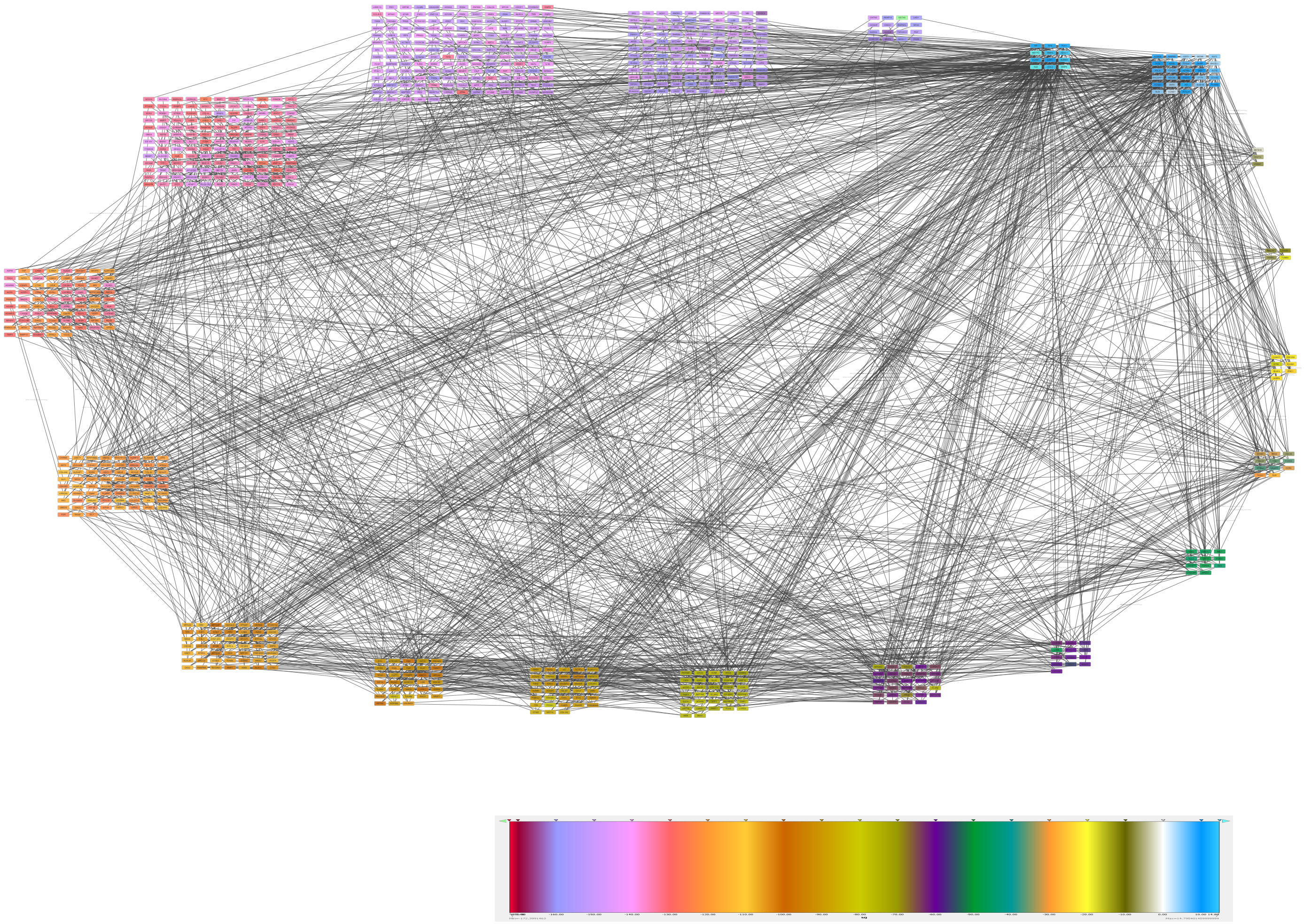

Figure S23. Local network entropy from MOD DYS PPIs network of Squamous cell carcinoma of the lung carcinogenic process. The multivariate entropy for the local networks of each node and colors assigned to each group are shown.

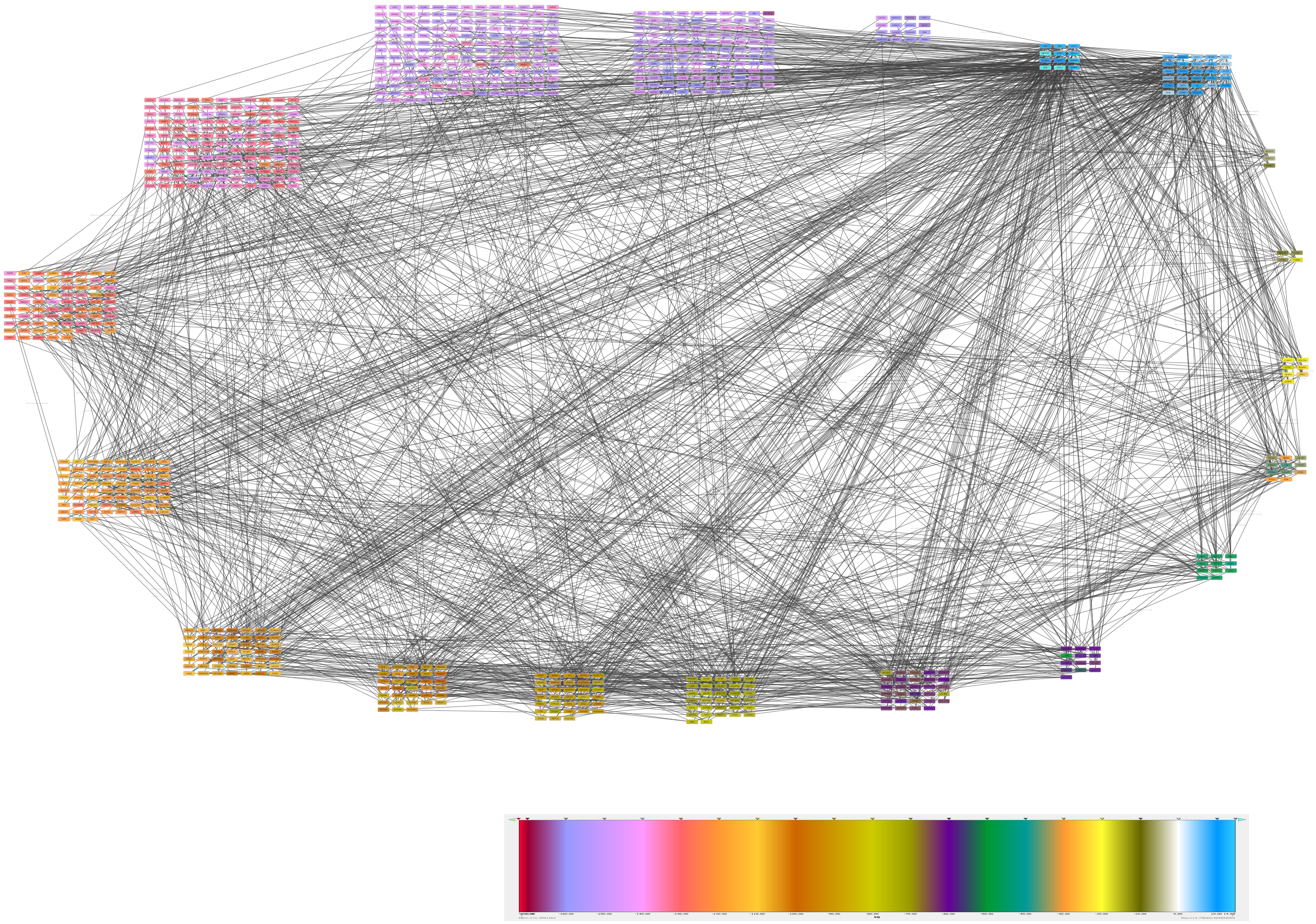

Figure S24. Local network entropy from SEV DYS PPIs network of Squamous cell carcinoma of the lung carcinogenic process. The multivariate entropy for the local networks of each node and colors assigned to each group are shown.

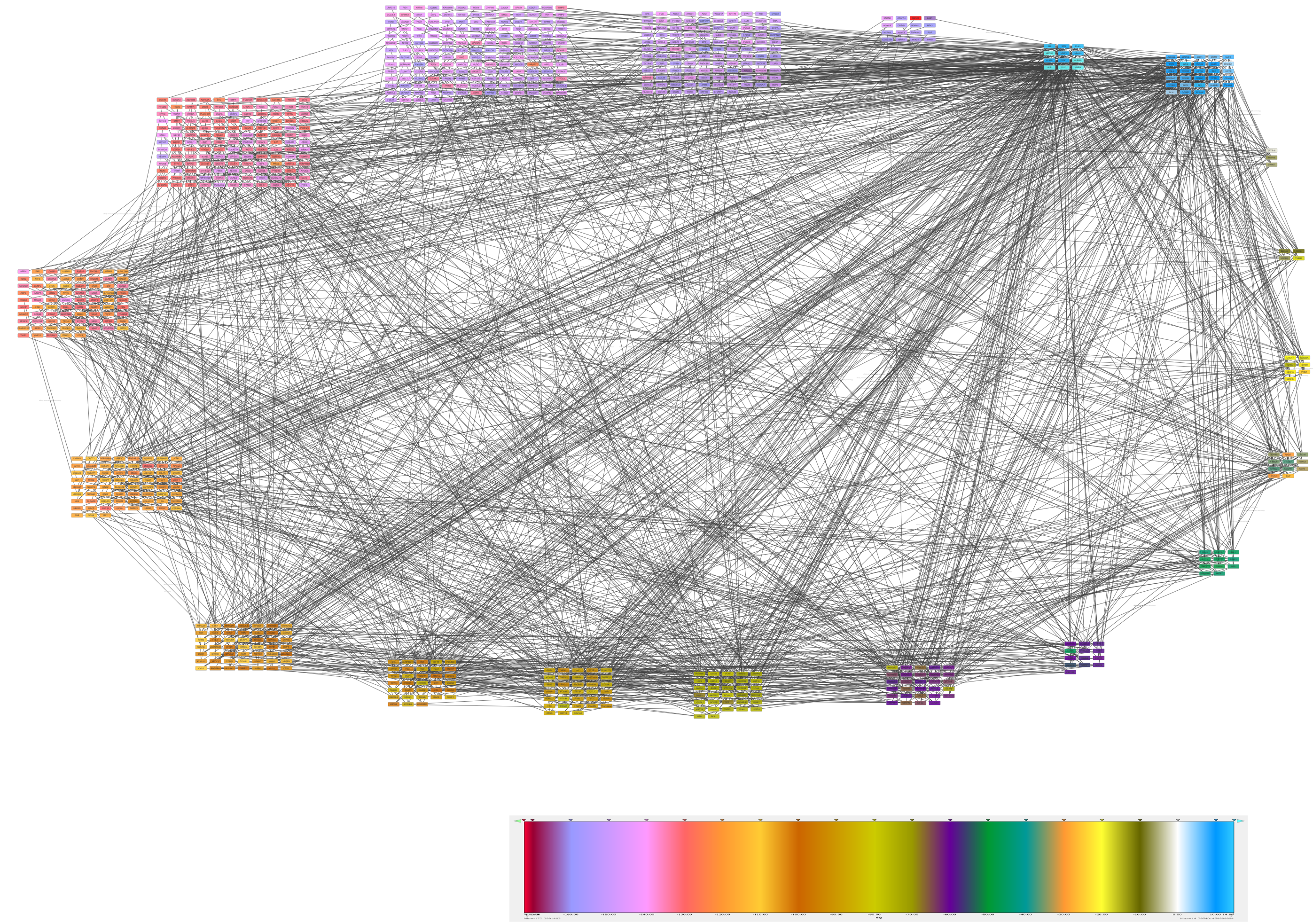

Figure S25.Local network entropy from C IN SITU PPIs network of Squamous cell carcinoma of the lung carcinogenic process. The multivariate entropy for the local networks of each node and colors assigned to each group are shown.

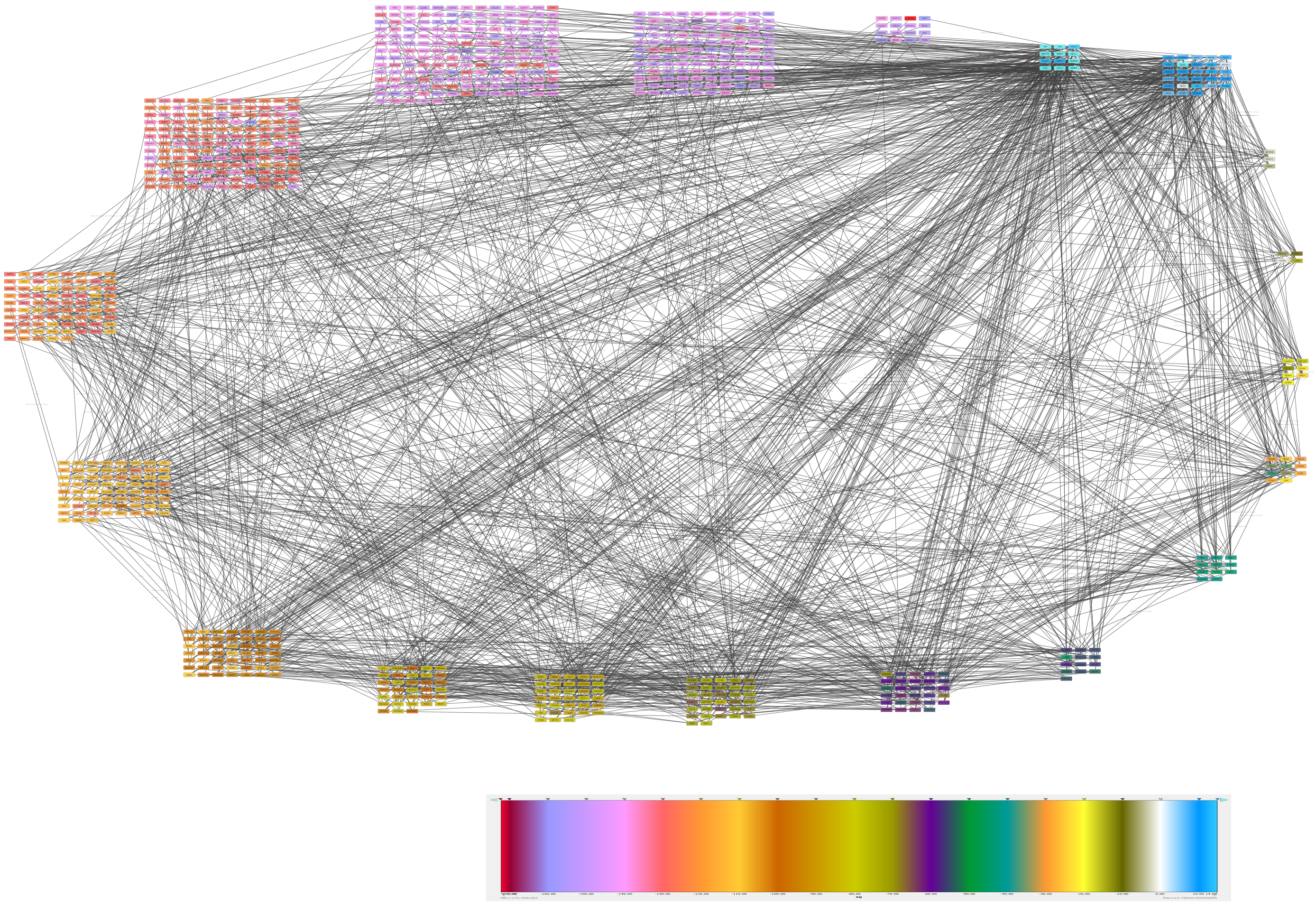

Figure S26.Local network entropy from SQ PPIs network of Squamous cell carcinoma of the lung carcinogenic process. The multivariate entropy for the local networks of each node and colors assigned to each group are shown.
